# Neuroimaging Advances in Parkinson's Disease and Atypical Parkinsonian Syndromes

**DOI:** 10.3389/fneur.2020.572976

**Published:** 2020-10-15

**Authors:** Usman Saeed, Anthony E. Lang, Mario Masellis

**Affiliations:** ^1^Hurvitz Brain Sciences Program, Sunnybrook Research Institute, Toronto, ON, Canada; ^2^Division of Neurology, Department of Medicine, University of Toronto, Toronto, ON, Canada; ^3^Edmond J Safra Program in Parkinson's Disease and the Morton and Gloria Shulman Movement Disorders Clinic, Toronto Western Hospital, University Health Network, Toronto, ON, Canada; ^4^L.C. Campbell Cognitive Neurology Research Unit, Sunnybrook Health Sciences Center, Toronto, ON, Canada; ^5^Cognitive and Movement Disorders Clinic, Sunnybrook Health Sciences Center, Toronto, ON, Canada

**Keywords:** biomarkers, Parkinson's disease (PD), atypical Parkinsonian syndromes, magnetic resonance imaging (MRI), single photon emission computed tomography (SPECT), diffusion-weighted imaging (DWI), transcranial sonography (TCS), positron emission tomography (PET)

## Abstract

Parkinson's disease (PD) and atypical Parkinsonian syndromes are progressive heterogeneous neurodegenerative diseases that share clinical characteristic of parkinsonism as a common feature, but are considered distinct clinicopathological disorders. Based on the predominant protein aggregates observed within the brain, these disorders are categorized as, (1) α-synucleinopathies, which include PD and other Lewy body spectrum disorders as well as multiple system atrophy, and (2) tauopathies, which comprise progressive supranuclear palsy and corticobasal degeneration. Although, great strides have been made in neurodegenerative disease research since the first medical description of PD in 1817 by James Parkinson, these disorders remain a major diagnostic and treatment challenge. A valid diagnosis at early disease stages is of paramount importance, as it can help accommodate differential prognostic and disease management approaches, enable the elucidation of reliable clinicopathological relationships ideally at prodromal stages, as well as facilitate the evaluation of novel therapeutics in clinical trials. However, the pursuit for early diagnosis in PD and atypical Parkinsonian syndromes is hindered by substantial clinical and pathological heterogeneity, which can influence disease presentation and progression. Therefore, reliable neuroimaging biomarkers are required in order to enhance diagnostic certainty and ensure more informed diagnostic decisions. In this article, an updated presentation of well-established and emerging neuroimaging biomarkers are reviewed from the following modalities: (1) structural magnetic resonance imaging (MRI), (2) diffusion-weighted and diffusion tensor MRI, (3) resting-state and task-based functional MRI, (4) proton magnetic resonance spectroscopy, (5) transcranial B-mode sonography for measuring substantia nigra and lentiform nucleus echogenicity, (6) single photon emission computed tomography for assessing the dopaminergic system and cerebral perfusion, and (7) positron emission tomography for quantifying nigrostriatal functions, glucose metabolism, amyloid, tau and α-synuclein molecular imaging, as well as neuroinflammation. Multiple biomarkers obtained from different neuroimaging modalities can provide distinct yet corroborative information on the underlying neurodegenerative processes. This integrative “multimodal approach” may prove superior to single modality-based methods. Indeed, owing to the international, multi-centered, collaborative research initiatives as well as refinements in neuroimaging technology that are currently underway, the upcoming decades will mark a pivotal and exciting era of further advancements in this field of neuroscience.

## Background

Parkinsonism defined by the presence of cardinal clinical motor features of rigidity, bradykinesia and tremor impacts the functioning of affected patients and can result in a significant loss of quality of life. Parkinson's disease (PD) is the most prevalent cause of neurodegenerative parkinsonism affecting more than 10 million individuals globally and bears a huge socioeconomic burden (1). The motor symptoms of PD, especially as seen in the early stages of the disease, are largely due to the loss of dopamine-producing neurons within the substantia nigra pars compacta (SNpc), whereas non-motor features that include olfactory and autonomic dysfunction, sleep disorders, psychiatric symptoms, depression, pain, fatigue, and cognitive impairment result from a more widespread neurodegeneration involving other neurotransmitter systems (2). On the basis of dopaminergic denervation, PD patients typically show a good response to levodopa. Conversely, the atypical Parkinsonian syndromes (PS) are a group of heterogenous neurodegenerative diseases that also present with parkinsonism, although they generally do not respond well to levodopa treatment and are considered distinct clinicopathological disorders.

Neurodegenerative diseases causing parkinsonism are categorized based on the predominant protein aggregates found within the brain, which are believed to be intimately involved in the underlying pathogenic mechanisms. Lewy body spectrum disorders (LBSD), including PD with and without cognitive impairment, Parkinson's disease dementia (PDD) and dementia with Lewy bodies (DLB) as well as multiple system atrophy (MSA) are classified as α-synucleinopathies due to the presence of misfolded α-synuclein aggregates. Progressive supranuclear palsy (PSP) and corticobasal degeneration (CBD) are categorized as tauopathies due to the preponderance of aggregated tau inclusions within the brain. With better understanding of these syndromes, the presence of “mixed” pathologies is increasingly being recognized. Indeed, concomitant aggregation of amyloid, tau, and α-synuclein proteins within the brain contribute to substantial heterogeneity in disease presentation and progression. The core neuropathological and clinical characterization of tauopathies and α-synucleinopathies are detailed in Table 1.

**Table 1:** Neuropathological and clinical characteristics of α-synucleinopathies and tauopathies.

**Neuropathological classifications**	**α-Synucleinopathies**		**Tauopathies**	
Neurodegenerative disorders	Lewy body spectrum disorders	Multiple system atrophy (MSA)	Progressive supranuclear palsy (PSP)	Corticobasal degeneration/syndrome (CBD/CBS)
Clinical subtypes	• Parkinson's disease (PD) • Parkinson's disease-mild cognitive impairment (PD-MCI) • Parkinson's disease dementia (PDD) • Dementia with Lewy bodies (DLB)	• MSA-parkinsonian (MSA-P) • MSA-cerebellar (MSA-C) • MSA with varying degrees of both parkinsonian and cerebellar features	• PSP-Richardson's syndrome (PSP-RS) • PSP-ocular motor • PSP-postural instability • PSP-parkinsonism (PSP-P) • PSP-frontal • PSP-progressive gait freezing • PSP-corticobasal syndrome • PSP-speech/language disorder • PSP-primary lateral sclerosis^a^ • PSP-cerebellar ataxia (PSP-C)^a^ (3, 4)	• CBS is clinically defined and is the most common manifestation of underlying CBD neuropathology, but it is not specific. Besides CBS, other presentations associated with CBD include progressive non-fluent aphasia, speech apraxia, posterior cortical atrophy, behavior variant frontotemporal lobal degeneration, PSP-like syndrome, among others (5, 6)
Core neuropathological features	• Intraneuronal fibrillar inclusions composed predominantly of misfolded α-synuclein protein within the cell body (Lewy bodies) and neuronal processes (Lewy neurites) (2) • Lewy pathology in Lewy body spectrum disorders may spread in a prion-like fashion beginning in the peripheral (e.g., enteric) nervous system and spreading to the lower brainstem (dorsal motor nucleus of the cranial nerve IX/X), followed by spread to the pons, midbrain, and subcortical regions (affecting dopaminergic neurons in the substantia nigra pars compacta, as in PD), eventually reaching the neocortex (as in DLB and PDD) (7). Whether this sequential spread is followed in all cases with Lewy body disorders is unclear • Based on autopsy, four Lewy-related disease subtypes have been identified: olfactory bulb only, amygdala predominant, brainstem, limbic (transitional), and diffuse neocortical (8) • Lewy pathology affecting the brainstem, limbic and neocortical regions is typically observed in DLB and PDD, whereas, brainstem Lewy pathology is predominant in PD (2, 8, 9) • Mixed AD pathology (amyloid and tau aggregates) is more frequently observed in DLB and PDD, which may lead to heterogeneity in the disease presentation (2, 10)	• Fibrillar cytoplasmic inclusions composed of misfolded α-synuclein protein within the oligodendrocytes (Papp-Lantos bodies) (11) • Loss of myelin, gliosis, axonal degeneration, and neuronal loss is observed in the olivopontocerebellar and striatonigral regions, hypothalamus, brainstem nuclei, as well as in the intermediolateral cell columns of the spinal cord (11, 12) • Pathogenesis of MSA may involve the interaction between p25α (a stabilizer of myelin integrity) and α-synuclein within the oligodendrocytes. This may initiate a cascade of events leading to neuroinflammation, loss of neurotrophic support, and neuronal dysfunction, eventually causing neurodegeneration in the striatonigral and olivopontocerebellar regions, as well as in the central autonomic pathways (11)	• Neurofibrillary tangles composed of hyperphosphorylated 4-repeat tau protein, neuropil threads, star-shaped tufted astrocytes, oligodendroglial coiled bodies, and gliosis are present, primarily in the basal ganglia, brainstem and diencephalon (4, 13, 14)	• Hyperphosphorylated 4-repeat tau protein within the cell bodies in the form of swollen, achromatic (ballooned) neurons, and in glial cells as astrocytic plaques. Gross neuronal loss is seen in an asymmetric fashion in the frontoparietal lobe (15) • The diagnosis of CBS is clinical in the absence of histopathological confirmation, and may include other pathologies in addition to the histopathologically-confirmed CBD, such as those of AD, PSP, Lewy bodies, and other tau-positive and tau-negative (primarily TDP-43 positive) forms of frontotemporal lobar degeneration (6)
Predominant clinical features	• Cardinal motor manifestations of PD consist of bradykinesia, rigidity, resting tremor, and postural and gait disturbances • Non-motor manifestations of PD may involve olfactory and autonomic dysfunction, sleep disorders, psychiatric symptoms, pain, depression, fatigue and cognitive impairment (2, 16) • Core clinical features of DLB include dementia together with cognitive and alertness fluctuations, recurrent visual hallucinations, features of parkinsonism, and/or rapid eye movement sleep behavior disorder. Other features, including repeated falls, dysautonomia (syncope, constipation, orthostatic hypotension, urinary incontinence), other psychiatric manifestations (delusion, apathy, depression), and hypersensitivity to neuroleptic medications may also be present in DLB and PDD (8, 12) • Distinction between PDD and DLB is based on the arbitrary “1 year” rule: dementia onset within 12 months of or contemporarily with motor symptoms qualifies as DLB, whereas parkinsonism must precede dementia by at minimum 1 year for PDD diagnosis. The MDS's diagnostic criteria for PD (17) do not apply this 1 year rule; patients can receive a diagnosis of PD even if they develop dementia before or within 1 year of parkinsonism • It is currently debatable whether PDD and DLB are distinct disorders with overlapping features or are the same disease with variability in clinical presentation (18)	• Non-motor features may precede the motor abnormalities, however, MSA is diagnosed and classified based on the predominant motor symptomology, as follows (19): 1) MSA-P, with parkinsonism as the predominant feature, which typically responds poorly to levodopa treatment, and is associated with marked striatonigral degeneration 2) MSA-C, with cerebellar syndrome and is associated with marked olivopontocerebellar atrophy 3) MSA patients showing varying degrees of both cerebellar and parkinsonian symptomology • Early dysautonomia is a non-motor characteristic of MSA, which includes orthostatic hypotension, erectile dysfunction, constipation, urinary incontinence/retention, respiratory stridor, and sweat gland dysfunction, where the latter may lead to thermoregulatory failure (11, 12, 19)	• Clinical manifestations are classified into four functional domains that include ocular motor dysfunction, postural instability, akinesia, and cognitive dysfunction (see below). Each of these domains contains features with varying degrees of certainty for the clinical diagnosis of PSP. Other supportive clinical and imaging features have also been incorporated in the new diagnostic criteria (3) • Ocular motor dysfunction includes features of vertical supranuclear gaze palsy, slow velocity of vertical saccades, and frequent macro square wave jerks or “eyelid opening apraxia” (3) • Postural instability includes symptoms of repeated unprovoked falls, tendency to fall on the pull test, and taking greater than two steps backward on the pull test, all within 3 years of symptom onset (3) • Akinesia includes the following features (with diminishing specificity for the diagnosis): (1) progressive gait freezing within 3 years, (2) parkinsonism that is akinetic-rigid, predominantly axial and levodopa resistant, and/or (3) parkinsonism accompanied by tremor and/or presence of asymmetry and/or levodopa responsiveness (3) • Cognitive dysfunction includes speech/language disorder, frontal cognitive or behavioral presentations, or corticobasal syndrome (3) • Unlike the classic syndrome PSP-RS, identified only in 24% of cases (20), the PSP-P variant presents with more prominent limb rigidity with bradykinesia and/or tremor with moderate response to levodopa in some patients, without early ocular or postural dysfunction (14)	• Classical syndrome associated with CBD includes basal ganglionic features, such as asymmetric limb rigidity, bradykinesia and dystonia, as well as cortical features, such as limb apraxia, aphasia, alien limb phenomenon and stimulus-sensitive myoclonus. Cognitive and behavioral changes may be seen early in the course of the disease (5, 10, 15) • Clinical findings are usually asymmetric, although this may not always be the case (15) • A large proportion of CBD cases initially present with behavioral or cognitive problems, whereas less than half initially present with motor involvement (15) • Clinicopathologic heterogeneity challenges the development of specific diagnostic criteria, as the pathology of CBD is predicted antemortem in only 25–56% of cases (15, 21)

*AD, Alzheimer's disease; CBD, corticobasal degeneration; CBS, corticobasal syndrome; DLB, dementia with Lewy bodies; MDS, Movement Disorder Society; MSA, multiple system atrophy; MSA-C, MSA-cerebellar type; MSA-P, MSA-parkinsonian type; PD, Parkinson's disease; PD-MCI, Parkinson's disease-mild cognitive impairment; PDD, Parkinson's disease dementia; PSP, progressive supranuclear palsy; PSP-C, PSP-cerebellar ataxia type; PSP-P, PSP-parkinsonism type; PSP-RS, PSP-Richardson's syndrome; TDP-43, transactive response DNA binding protein-43 kDa; a = these phenotypes are not included in the new diagnostic criteria due to low specificity for PSP (3, 4)*.

Our understanding of PD and atypical PS has been significantly enhanced by methodological and analytical improvements in *in vivo* neuroimaging techniques. Neuroimaging can be used to: (1) identify disease-specific structural and functional biomarkers, some of which have been incorporated into the diagnostic criteria and may serve to enhance diagnostic confidence, (2) rule out unrelated abnormalities (e.g., neoplasms, strokes, extensive cerebrovascular pathology) as primary or contributory cause of the symptoms, (3) validate promising prodromal biomarkers for diagnostic purposes, which may also assist in patient recruitment or sample enrichment for therapeutic trials, (4) quantify whole-brain or regional burden of misfolded neuropathological molecules (e.g., amyloid, tau, or α-synuclein and their co-aggregation) as well as other physiological processes (e.g., neuroinflammation), and (5) study disease progression over time or in response to therapeutic interventions via the evaluation of neuroimaging-based secondary outcome measures. Several neuroimaging modalities have been developed and applied to Parkinsonian disorders, each providing distinct information on the underlying brain disorders. An overview of the common neuroimaging techniques is summarized in Table 2.

**Table 2:** An overview of the common neuroimaging modalities discussed in this article.

**Neuroimaging modality**	**Measures**	**Description**
1) MRI		
• Structural MRI	Atrophy pattern, volume, cortical thickness, ventricular enlargement, white matter hyperintensities, magnetic inhomogeneity effects	Visualization and quantification of brain's structural changes using regions-of-interest or whole-brain approaches. The following MRI sequences are commonly used: T1, T2, T2*, R2* (R2*= 1/T2*)-weighted, susceptibility-weighted, proton-density-weighted, fluid-attenuated inversion recovery, and neuromelanin-sensitive sequences
• Diffusion-weighted and diffusion tensor MRI	Mean diffusivity (*D*), radial diffusivity, axial diffusivity, fractional anisotropy (FA)	Measures brain's microstructural integrity by assessing the movement of water molecules. Damage to white matter tracts restricts the directional movement of water molecules resulting in increased *D* and decreased FA
• Functional MRI	Functional connectivity, change or correlation in blood-oxygen-level-dependent signal	Evaluates neuronal activity in the brain by measuring the transient variations in the blood flow and whether this variation correlates in functionally-connected regions. Functional MRI can be utilized under a variety of experimental paradigms (e.g., task-based vs. control conditions) or under resting-state conditions
2) Proton magnetic resonance spectroscopy	Abundance of metabolites	Estimates the relative concentrations of proton-containing metabolites in the brain. In neurodegenerative disorders, the following metabolites are commonly assessed: N-acetyl aspartate, choline-containing compounds (including free choline, phosphorylcholine and glycerophosphorylcholine), myo-inositol, and creatine
3) Transcranial B-mode sonography	Echogenicity	Uses an ultrasound machine to measure the echogenicity of brain tissues or structures (e.g., substantia nigra, lentiform nucleus, basal ganglia) through the intact cranium. Limitations include the lack of sufficient bone window rendering this technique infeasible in some subjects, and the need for trained examiners for reliable detection and measurement of the imaged features
4) SPECT		
• Striatal presynaptic dopaminergic system	Dopamine transporter (DAT) density	Evaluates nigrostriatal integrity by measuring the density of DATs—sodium-coupled transmembrane protein located at the presynaptic nigrostriatal terminals that mediate the reuptake of dopamine from the synaptic cleft. The most widely used radioligand for measuring DAT density has been ^123^I-FP-CIT
• Striatal postsynaptic dopaminergic system	Dopamine D2 receptor density	Evaluates nigrostriatal integrity by measuring the density of striatal postsynaptic dopamine D2 receptors (G-protein-coupled) using radioligands, such as ^123^I-IBZM and ^123^I-IBF
• Cerebral perfusion	Metabolic activity (by measuring changes in the cerebral blood flow)	Provides a measure of the perfusion and metabolic status of the brain tissues, which can be imaged using lipophilic radiotracers, such as ^99m^Tc-ECD, ^99m^Tc-HMPAO and ^123^I-IMP
5) PET		
• Striatal presynaptic dopaminergic system	Aromatic amino acid decarboxylase (AADC) activity, vesicular monoamine transporter type 2 (VMAT2) density	Density of presynaptic nigrostriatal axons can be assessed using ^18^F-dopa radiotracer for PET. Specifically, the activity of AADC protein is evaluated, which converts ^18^F-dopa into ^18^F-dopamine, providing an approximation of dopaminergic storage pool. Presynaptic monoaminergic system can be assessed using ^11^C-DTBZ or ^18^F-labeled analogs (e.g., ^18^F-AV133), which binds to VMAT2—a presynaptic transmembrane protein essential for packaging and storing monoamines (which include dopamine) into synaptic vesicles
• Striatal postsynaptic dopaminergic system	Dopamine D2 receptor density	Density of postsynaptic dopamine D2 receptors can be examined using ^11^C-raclopride radiotracer for PET
• Cholinergic system	Acetylcholine esterase activity	Integrity of cholinergic neurons (e.g., in the nucleus basalis of Meynert) can be assessed using PET tracers, ^11^C-NMP4A or ^11^C-PMP, which measure the activity of acetylcholine esterase
• Serotonergic system	Serotonin transporter, 5-HT1A receptor sites on serotonergic neurons	Serotonergic function can be evaluated by targeting, (1) 5-HT1A autoreceptors found on serotonergic cell bodies in the median raphe and on pyramidal cells in the limbic cortex using ^11^C-WAY100635 or ^18^F-MPPF PET, or (2) serotonin transporters found in the brainstem and cortex using ^11^C-DASB PET as well as other SPECT tracers
• Noradrenaline system	Noradrenaline transporter	Noradrenaline neurotransmitter dysfunction may be quantified using ^11^C-RTI-32 ligand, which binds to both dopamine and noradrenaline transporters
• Glucose metabolism	Metabolic activity (by measuring changes in the glucose consumption)	Cerebral glucose metabolism can be measured using ^18^F-labeled fluorodeoxyglucose (^18^F-FDG) radiotracer. Decreased ^18^F-FDG uptake on PET is indicative of lower regional tissue metabolism of glucose
• Amyloid	Amyloid pathology	Cerebral amyloidopathy has commonly been evaluated on PET using Pittsburgh compound B (^11^C-PIB)—a ^11^C-labeled thioflavin analog with a half-life of 20 min—as well as using other ^18^F-labeled radioligands that have a relatively longer half-life (~110 min), such as ^18^F-florbetapir, ^18^F-florbetaben, and ^18^F-flutemetamol
• Tau	Tau pathology	Cerebral tauopathy can be visualized using PET radiotracers including ^18^F-AV-1451 (known as ^18^F-flortaucipir or ^18^F-T807), ^18^F-FDDNP, ^18^F-THK523, ^18^F-THK5351, ^18^F-THK5105, and ^11^C-PBB3
• α-Synuclein	α-Synuclein pathology	Several PET radiolabeled probes for imaging cerebral α-synucleinopathy have been explored (phenothiazine, indolinone, indolinone-diene and chalcone analogs); however, none have been approved for use in clinical and research settings
• Neuroinflammation	Microglia-mediated inflammatory processes	Neuroinflammation can be assessed using PET radiotracers including ^11^C-PK11195, ^11^C-PBR28 and ^18^F-FEPPA, which detect the upregulation of TSPO protein located on the outer mitochondrial membrane of microglia

*AADC, aromatic amino acid decarboxylase; D, mean diffusivity; DAT, dopamine transporter; FA, fractional anisotropy; MRI, magnetic resonance imaging; PET, positron emission tomography; SPECT, single photon emission computed tomography; TSPO, translocator protein-18 kDa; VMAT2, vesicular monoamine transporter type 2*.

In this review, a comprehensive presentation of the current and emerging biomarkers from multiple neuroimaging modalities in PD and atypical PS will be undertaken with emphasis on some of the distinguishing characteristics.

## Search Strategy

The literature search was performed on the PubMed database using the following disease-specific keywords: “Parkinson^*^,” “Lewy^*^,” “multiple system atrophy,” “corticobasal degeneration,” “progressive supranuclear palsy” —together with one of the modality-specific keywords: “magnetic resonance imaging,” “positron emission tomography,” “single-photon emission computed tomography,” “diffusion tensor,” “diffusion-weighted,” “proton spectroscopy,” and “transcranial sonography.” Acronyms, e.g., “PET” for “positron emission tomography,” were entered as appropriate. The literature search was restricted to articles written in English, and published between January 1, 1995 and December 31, 2019. All abstracts were screened for relevance. The most pertinent articles were then read and discussed.

## Structural Neuroimaging in Parkinsonian Disorders

### Parkinson's Disease (PD)

#### Structural Magnetic Resonance Imaging in PD

**Figure 1:**
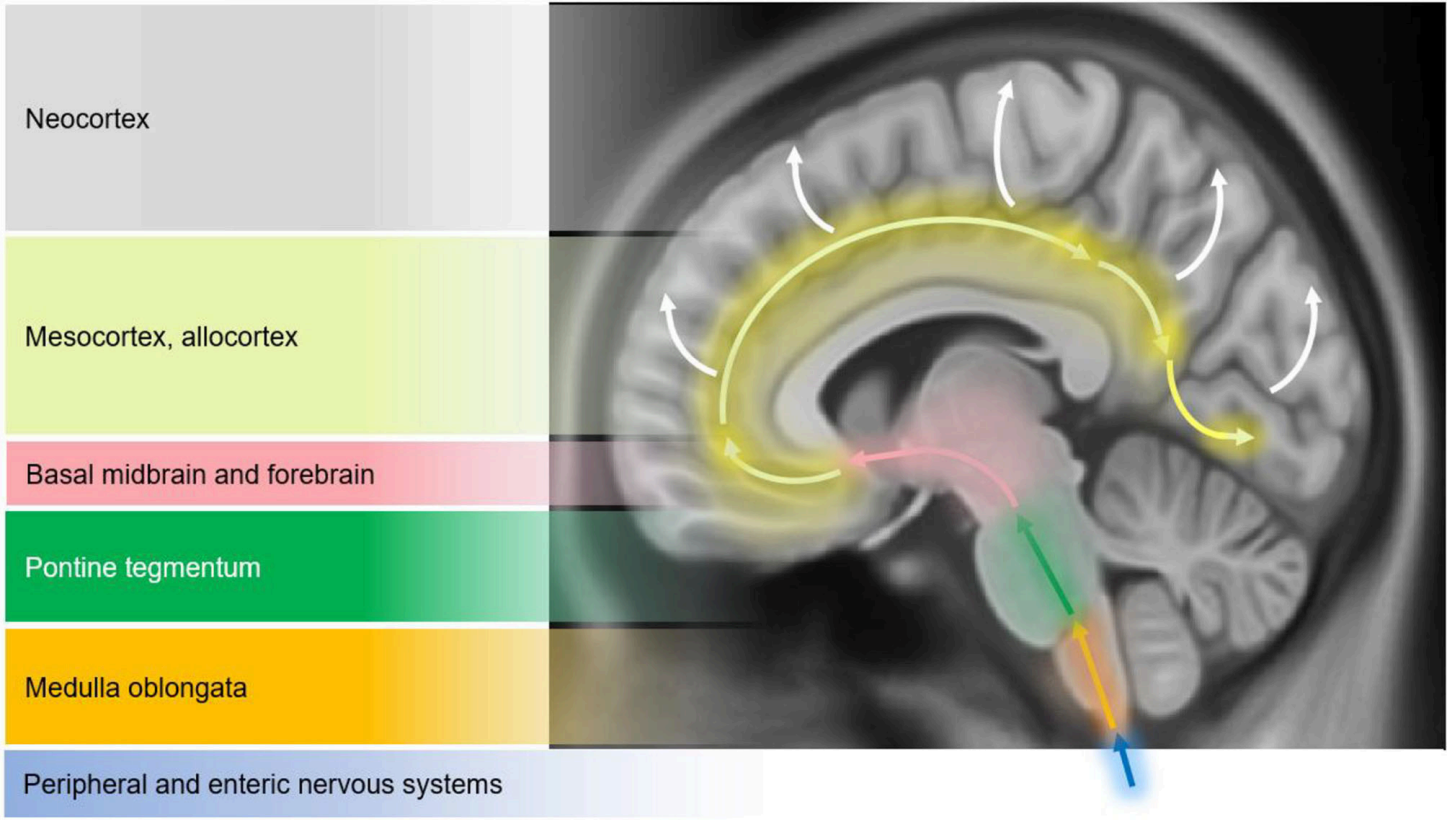
Schematic diagram illustrating the progression of α-synuclein pathology (Lewy bodies and Lewy neurites) in Parkinson's disease (PD), as proposed by Braak et al. (7). According to the Braak model, α-synuclein pathology in the brain spreads caudo-rostrally in a characteristic pattern starting in stage I and II in the lower brainstem regions of medulla oblongata and pons (dorsal motor nucleus of the cranial nerve IX/X, raphe nuclei, gigantocellular reticular nucleus, and coeruleus-subcoeruleus complex). In stages III and IV, α-synucleinopathy spreads further to the susceptible regions of the midbrain (e.g., dopaminergic neurons in the substantia nigra pars compacta), forebrain (e.g., hypothalamus, thalamus, and limbic system), as well as involving some of the cortical regions in the temporal mesocortex (transentorhinal region) and allocortex. In the last two stages (V and VI), α-synuclein pathology reaches the neocortex contributing to cognitive dysfunction (as seen in dementia with Lewy bodies and PD dementia). It is hypothesized that the initiation site of α-synuclein pathology may be outside the central nervous system (CNS), probably beginning in the peripheral (enteric) nervous system and gaining access to the CNS through retrograde transport mechanisms in a prion-like fashion. Whether this sequential spread of α-synuclein pathology as proposed by the Braak model is followed in all cases in Lewy body spectrum disorders is less clear. Figure adapted from Visanji et al. (32), under the Creative Commons Attribution License (https://creativecommons.org/licenses/by/2.0/).

**Figure 2:**
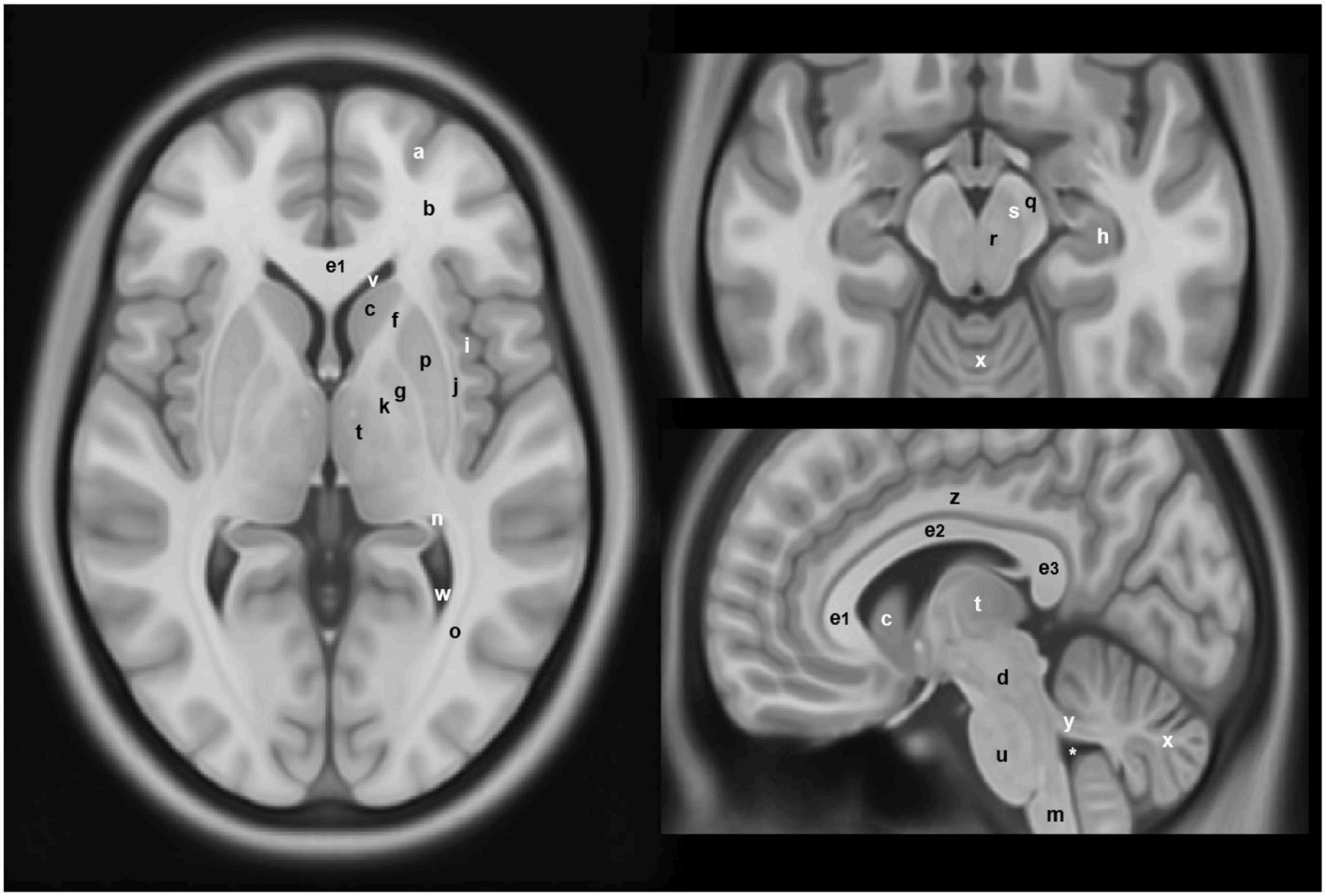
Anatomical locations of some of the structures and regions important in Parkinson's disease and atypical Parkinsonian syndromes, highlighted on a standard averaged T1-weighted MNI template for normal population. Labeling: a = cerebral gray matter (frontal lobe), b = cerebral white matter (frontal lobe), c = head of caudate nucleus, d = midbrain, e1 = genu of corpus callosum, e2 = body of corpus callosum, e3 = splenium of corpus callosum, f = anterior limb of internal capsule, g = globus pallidus, h = hippocampus, i = insular cortex, j = claustrum, k = posterior limb of internal capsule, m = medulla oblongata, n = tail of caudate nucleus, o = optic radiation, p = putamen, q = crus cerebri (anterior portion of cerebral peduncle), r = red nucleus, s = substantia nigra, t = thalamus, u = pons, v = anterior horn of lateral ventricle, w = posterior horn of lateral ventricle, x = cerebellum, y = superior cerebellar peduncle, z = cingulate gyrus, * = fourth ventricle. Note: p and g together constitute the lentiform nucleus; c and p together constitute the dorsal striatum. The template was obtained from McConnell Brain Imaging Center, Montreal Neurological Institute, McGill University Copyright 1993–2004 Fonov et al. (33).

Structural changes on conventional magnetic resonance imaging (MRI) are minimal and less apparent, especially in early stages of PD (10). A six-stage model depicting the sequential progression of α-synuclein pathology in PD has been proposed by Braak et al. (7) (Figure 1). As per this “gut to brain” transmission model, neuroimaging studies may predominantly observe brainstem and subcortical involvement in early PD stages with greater cortical involvement in late stage PD as well as in PD-MCI, PDD and DLB cases. Compared to normal controls, voxel-based morphometry (VBM) studies in PD may identify atrophy in the basal ganglia (22) (as consistent with nigrostriatal degeneration and consequential dysfunction in the basal ganglia thalamocortical circuit), frontal lobe (23), and non-specifically in the right hippocampus, left anterior cingulate and superior temporal gyri (24). Although inconsistently shown, cortical thinning in the orbitofrontal, ventrolateral prefrontal, and occipitoparietal cortical regions has been identified in PD, along with volumetric reductions in the caudate and putamen (25, 26). Some studies do not observe appreciable differences on conventional MRI compared to controls (27, 28). In PD patients with olfactory disturbances, reduced volume in the olfactory bulb and tract was evident vs. MSA and controls (29). In PD patients with respiratory dysfunction, gray matter (GM) atrophy was reported in the left parahippocampal formation, right fusiform gyrus, right cerebellum crus, and left postcentral gyri compared to PD with normal pulmonary functions (30). Freezing of gait symptomatology in PD was associated with posterior GM atrophy (specifically, left cuneus, precuneus, lingual gyrus, and posterior cingulate cortex) (27). In advanced PD, atrophy in the subcortical GM structures was found to be more pronounced vs. those in early PD stages (31) (Figure 2).

**Figure 3:**
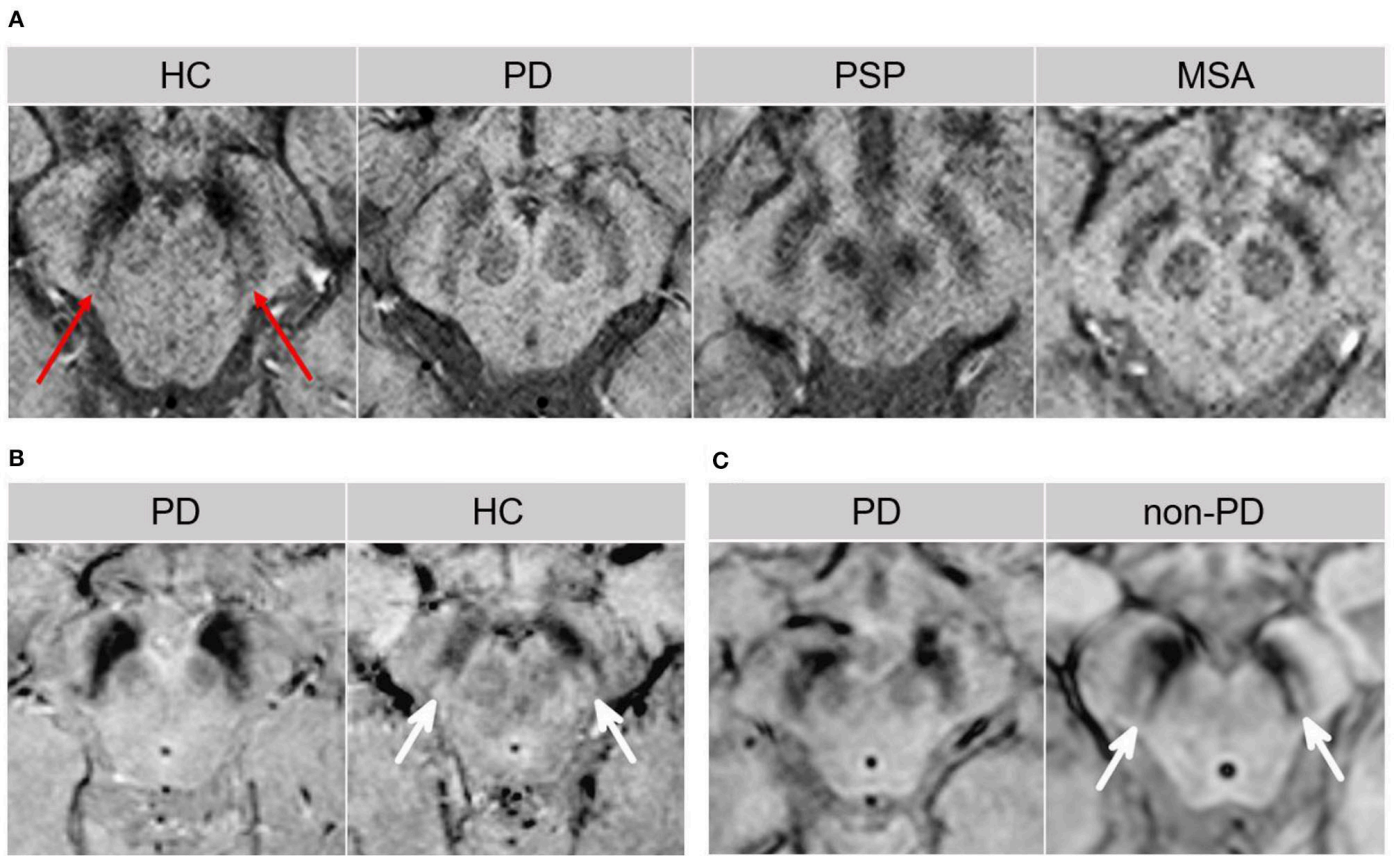
The “swallow tail” sign. All MRI presented above are taken at the level of substantia nigra in the midbrain. **(A)** Susceptibility-weighted MRI depicting dorsolateral nigral hyperintensity (the “swallow tail” sign, red arrows) in a healthy control. Loss of dorsolateral nigral hyperintensity can be seen in PD and may even be seen in some PSP and MSA cases on susceptibility-weighted MRI. **(B)** High resolution susceptibility-weighted MRI (gradient echo-echo planar imaging sequence, magnitude image) is shown for a PD patient and a control. **(C)** High resolution T2*/susceptibility-weighted MRI (multi-shot fast field echo-echo planar imaging sequence) is shown for a PD patient and a non-PD case who was diagnosed with aneurysmal subarachnoid hemorrhage. In both **(B,C)**, loss of dorsolateral nigral hyperintensity (white arrows) corresponding to nigrosome-1 can be seen in PD as compared to control and a non-PD subject. **(A)** was adapted from Chougar et al. (40), and **(B,C)** were adapted from Schwarz et al. (39), under the Creative Commons Attribution License (https://creativecommons.org/licenses/by/4.0/). HC, healthy control; MSA, multiple system atrophy; PD, Parkinson's disease; PSP, progressive supranuclear palsy.

Changes within the SN may emerge as promising early diagnostic biomarkers of PD (10). Within the SNpc, calbindin-negative pockets termed “nigrosomes” are observed (34). The greatest loss in neuromelanin containing neurons takes place in the nigrosome-1, which is located in the caudal and mediolateral portion of SNpc (35). Nigrosome-1 shows a significant loss of hyperintensity on T2^*^ and neuromelanin-sensitive MRI in PD, probably caused by decreased neuromelanin, increased iron content, or loss of paramagnetic neuromelanin–iron complexes (36–38). On susceptibility-weighted imaging (modality of choice), healthy nigrosome-1 and the surrounding neuroanatomy of the dorsolateral SN may appear as the tail of a swallow bird (“swallow-tail” sign). Loss of this feature in PD vs. controls may assist in the differential diagnosis (sensitivity 80%, specificity 89%) (39, 40) (Figure 3). Finally, neuromelanin-sensitive MRI has shown promise in the differentiation of PD from essential tremor and normal controls (37, 41), and this technique may prove invaluable as a marker of disease progression in PD.

In PD patients with polysomnography-confirmed rapid eye movement sleep behavior disorder (RBD), cortical thinning was reported in the right perisylvian and inferior temporal cortices together with shape changes in the putamen compared to PD without RBD (42). Likewise, decreased volume in the right putamen correlating with RBD symptom severity was identified in PD with RBD vs. those without (43). On susceptibility-weighted imaging, loss of dorsal nigral hyperintensity (corresponding to nigrosome-1) was observed in ~25% of patients with idiopathic RBD, which associated with lower putaminal dopamine transporter (DAT) binding on single-photon emission computed tomography (SPECT) (44). This may suggest nigrosome-1 degeneration in some of the RBD cases, likely those at risk of progression to PD (44). These studies propose a link between PD, the presence of RBD, and greater neurodegeneration especially in subcortical structures.

Volumetric changes in the SN have been inconsistently reported in PD vs. controls (45–48). A high resolution examination of structural alterations in the SN is possible using ultra-high-field MRI (49). For example, susceptibility-weighted imaging at 7T permitted the visualization of the anatomical layers of the SN, allowing excellent discrimination between PD and controls (sensitivity 100%, specificity 96.2%) (50). Correlations between motor symptoms and increased R2^*^ (apparent transverse relaxation rate, R2^*^ = 1/T2^*^) values in the SN have also been reported in PD (46, 51–53), which may reflect ferritin-induced magnetic field inhomogeneities. A recent study applied quantitative susceptibility mapping in PD to evaluate the magnetostatic alterations caused by changes in the iron distribution across the whole brain (54). The apparent magnetic susceptibility was found to be elevated in the dorsal and ventral SN, rostral pontine regions, and the cortex (primarily in the temporal paralimbic, prefrontal, and occipitoparietal regions) (54). Conversely, reduced magnetic susceptibility was detected in the normally iron-rich cerebellar region of the dentate nucleus suggesting decreased iron content (54). Interestingly, the striatum as well as the primary motor and somatosensory fields were spared (54). Future research will highlight whether iron accumulation is the consequence or cause of neurodegenerative changes associated with PD.

#### Diffusion-Weighted and Diffusion Tensor Imaging in PD

Reduced fractional anisotropy (FA) in the SN is commonly observed in PD (55, 56). Increased mean diffusivity (*D*) in the olfactory tracts and decreased FA in the anterior olfactory structures have been reported (57, 58), as consistent with neuronal loss observed in these regions (29). Significant differences in *D* or FA may not be evident in early PD due to milder neurodegeneration (59, 60). Combined analysis of diffusion parameters and apparent transverse relaxation rates was found to be superior in differentiating PD vs. MSA-P (sensitivity 97%, specificity 100%, positive predictive value [PPV] 100%, negative predictive value [NPV] 93%), and PD vs. MSA-P/PSP group (sensitivity 86%, specificity 87%, PPV 88%, NPV 84%) (61). In SNpc (delineated using a probabilistic atlas based on neuromelanin sensitive imaging), asymmetric alterations predominantly in the diffusion metrices rather than anisotropy were observed (62).

PD-MCI cases showed increased *D* as well as lower GM volume in the nucleus basalis of Meynert vs. cognitively normal PD (60). The degeneration of this cholinergic structure may identify patients at risk of more significant cognitive decline and dementia (60). Another study delineated the cortical projections of five corpus callosum segments and highlighted the role of callosal white matter (WM) abnormalities in cognitive dysfunction, which can occur via disruption of interhemispheric information transfer along callosal-cortical projections (63). In PD, increased axial diffusivity was identified in the three anterior callosal segments (projecting to prefrontal, premotor, motor, and supplementary motor cortices) vs. controls (63). Cognitive performance strongly related to diffusion tensor imaging (DTI) metrics in the most anterior (projecting to prefrontal cortex) and most posterior callosal sections (projecting to parietal, temporal, and occipital cortex), which may contribute to “fronto-striatal” and “posterior cortical” types of cognitive deficits seen in PD, respectively (63).

Free water was elevated in PD in posterior SN vs. controls (52, 64, 65). Increase in free water in posterior SN over time was observed in *de novo* PD patients, which was associated with motor severity and putaminal DAT SPECT binding (64). Given the posterior-to-anterior pattern of degenerative changes reported in SN, elevated free-water may also be observed in the anterior SN (66), especially in late-stage idiopathic PD (67). In contrast, free-water-corrected FA values were found to be unchanged in PD vs. controls, as assessed using the bi-tensor diffusion model (66, 68). Free-water and free-water-corrected FA values beyond SN and from multiple brain regions may help distinguish PD, MSA, and PSP cases from each other (66).

#### Proton Magnetic Resonance Spectroscopy in PD

Reduced N-acetyl aspartate/creatine (NAA/Cr) ratios in the SN have been observed in PD vs. controls, which were shown to correlate with disease severity (69, 70). Lowered NAA or NAA/Cr values have also been detected in other regions, including the lentiform nucleus (LN) (comprises of putamen and globus pallidus; basic anatomy presented in Figure 2), temporoparietal and posterior cingulate cortex, and pre-supplementary motor area vs. controls (71–74). However, the correlation between NAA/Cr ratios in these regions with disease severity or duration is inconsistently reported (72, 73). Another study compared the NAA/Cr ratios in the rostral and caudal SN, and found lower values in the rostral region in PD, whereas this pattern was inverted in the atypical PS group and controls (75). In tremor-dominant PD, reduced NAA/Cr and Choline/Cr values were detected in the thalamus vs. patients with essential tremor presenting with resting tremor (76). Cerebellar NAA/Cr and NAA/myo-inositol ratios were smaller in the atypical PS group compared to PD and controls (77). One study has also suggested the utility of proton magnetic resonance spectroscopy for the evaluation of treatment efficacy in PD (78). Specifically, the putaminal levels of myo-inositol, total Cr, and total NAA metabolites were reduced in the drug-off condition in PD vs. healthy controls. The administration of levodopa resulted in the restoration of total Cr and total NAA levels suggesting therapeutic responsiveness (78).

### Lewy Body Spectrum Disorders (LBSD)

#### Structural Magnetic Resonance Imaging in LBSD

In DLB and PDD, conventional MRI typically shows variable changes. Compared to controls, VBM studies in PDD have identified a diffuse pattern of cortical atrophy involving the occipital, temporal, right frontal, and left parietal lobe (23), as well as atrophy involving the putamen, hippocampus, parahippocampal region, anterior cingulate gyrus, nucleus accumbens and the thalamic nuclei (24). Although inconsistently reported (23), a greater cortical loss in the temporal, occipital, and parietal lobes was noted in DLB vs. PDD (79). Indeed, pathological heterogeneity evident in these two closely related α-synucleinopathies is in part responsible for variable findings. Compared to PD, atrophy in the occipital lobe and entorhinal cortex may help differentiate PDD (23, 80).

The relative preservation of total hippocampus compared to AD is a supportive diagnostic feature of DLB (8). Within the hippocampus, the CA1 subfield shows preservation (81), which aligns with the histopathological evidence showing Lewy body aggregates and neuronal loss largely localized to the CA2/3 subfields in DLB cases (81, 82). Conversely, atrophy in the CA1 subfield is indicative of neurofibrillary tangle pathology as evident in AD. Hippocampal atrophy is in fact observed in LBSD, controls and AD in a characteristic pattern [controls < PD < PDD/DLB < AD (9, 83)] and the extent of atrophy aligns with the underlying concomitant AD-type pathology (9). Smaller caudate and putaminal volumes have also been reported in PD and DLB vs. AD and controls, albeit inconsistently (26, 84). WM hyperintensities are more prevalent in PDD and DLB vs. PD and controls (85).

PD-MCI patients may show greater cortical thinning in temporoparietal, occipital, and supplementary motor area vs. cognitively-normal PD (86). A recent meta-analysis evaluating VBM studies identified pronounced GM atrophy in the left anterior insula in PD-MCI vs. cognitively-normal PD cases (87). Longitudinal cognitive decline in PD was associated with an AD-like pattern of cerebral atrophy at baseline, underscoring the contribution of the hippocampus and temporoparietal cortex in the cognitive sequelae of PD (88).

The ε4-allele of the apolipoprotein E gene (*APOE*-ε4) is a shared risk factor for AD, PDD, and DLB disorders (89). Therefore, the identification of neuroimaging and cognitive endophenotypes of *APOE*-ε4 irrespective of the clinical diagnosis has been pursued (9). Recent investigations indicate that *APOE*-ε4 is related to hippocampal atrophy along with learning and memory performance in DLB as well as across the AD/DLB spectrum, implicating *APOE*-ε4-associated shared neurodegenerative mechanisms across these disorders (9). Similarly, MRI-derived WM hyperintensity burden was inversely related to learning/memory, attention/executive and language performances in *APOE*-ε4 carriers across the AD/DLB spectrum (90). In addition to the *APOE*-ε4's influence on amyloidopathy, these results are consistent with the emerging evidence indicating an independent role of *APOE*-ε4 in modulating α-synucleinopathy in the brain (91).

#### Diffusion-Weighted and Diffusion Tensor Imaging in LBSD

In DLB vs. controls, diffusion imaging studies reveal *D* and FA abnormalities in the corpus callosum, pericallosal regions, caudate nucleus (suggestive of nigrostriatal involvement), amygdala, inferior longitudinal fasciculus, precuneus, as well as in the frontal, parietal, and occipital WM with milder involvement of the temporal lobe early in the disease (92–94). In DLB, elevated *D* in the amygdala (a region preferentially affected by Lewy pathology) was observed in the absence of significant GM density changes, probably suggesting microvacuolation or spongiosis as one of the possible mechanisms (93). Increased *D* in the longitudinal fasciculus was identified in DLB patients with hallucinations vs. those without (93). In PDD, reduced FA in the bilateral posterior cingulate bundle was reported vs. PD (95). Compared to AD, decreased FA was noted in the pons and left thalamus in DLB (96). However, given the significant pathological overlap between AD and DLB, diffusion imaging studies may not reveal consistent findings, particularly in subjects at advanced disease stages (94).

#### Proton Magnetic Resonance Spectroscopy in LBSD

In DLB, lower NAA/Cr ratios in the bilateral hippocampus were evident relative to controls (97), albeit milder than typically seen in AD. Lower NAA/Cr ratios were observed in the posterior cingulate gyrus in PDD vs. cognitively normal PD patients (98).

### Multiple System Atrophy (MSA)

#### Magnetic Resonance Imaging in MSA

**Figure 4:**
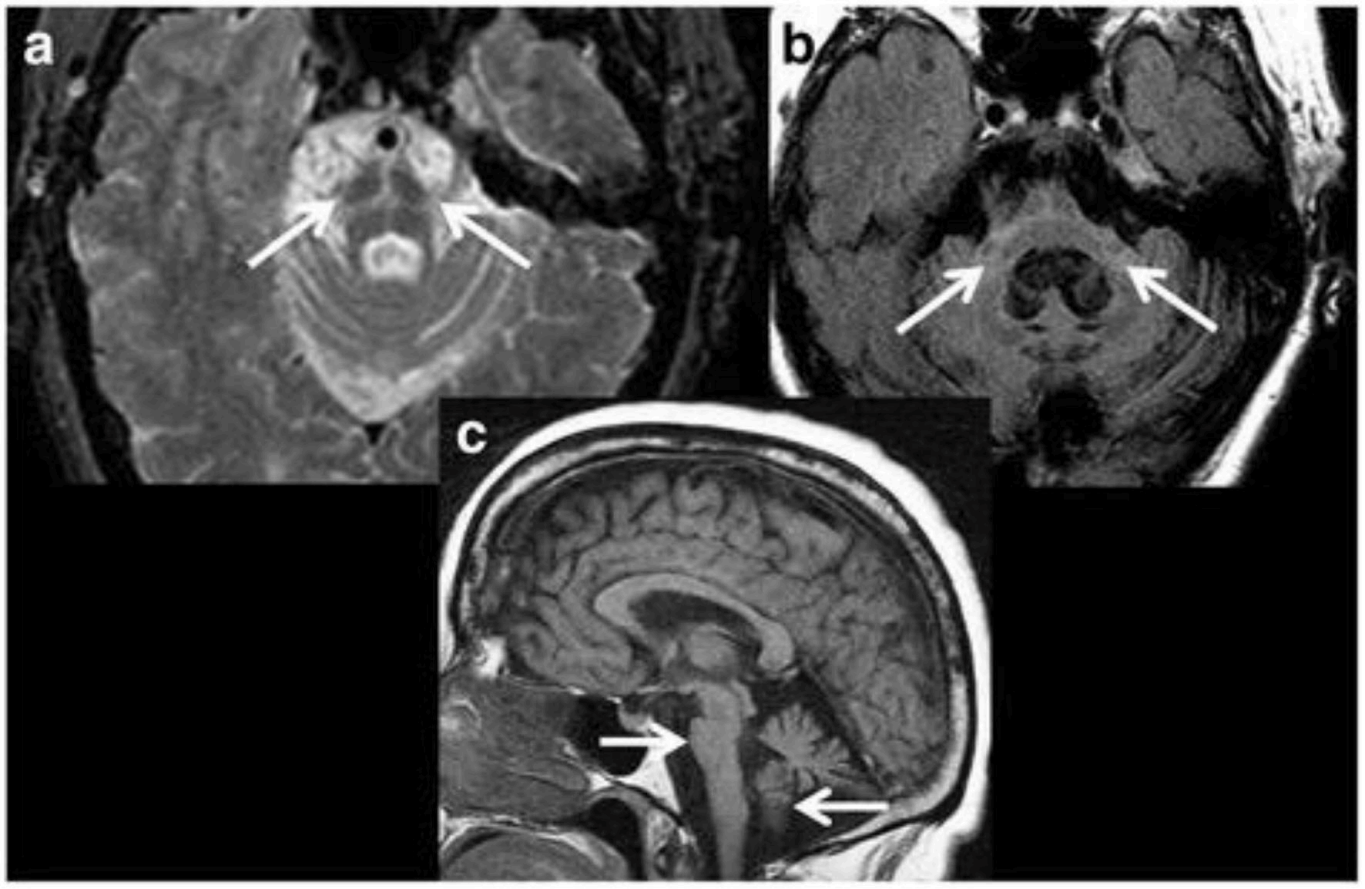
Magnetic resonance imaging of a patient clinically-diagnosed with multiple system atrophy (cerebellar type). **(a)** Axial proton density weighted sequence is presented at the level of pons, which shows cruciform pontine T2 hyperintensity as consistent with the “hot cross bun” sign, resulting from selective susceptibility of the pontocerebellar tract in multiple system atrophy (cerebellar type). In addition, disproportionate atrophy of the pons and partially visible cerebellar hemispheres are also apparent. **(b)** Axial fluid-attenuated inversion recovery (FLAIR) sequence is presented with cruciform T2 hyperintensity within the pons and middle cerebellar peduncles (i.e., “middle cerebellar peduncle” sign) along with marked atrophy. In addition, cerebellar hemispheric and vermian atrophy is evident with *ex vacuo* dilatation of the fourth ventricle. **(c)** Sagittal T1-weighted sequence is presented showing disproportionate atrophy of the brainstem and cerebellar vermis. Figure reproduced from Saeed et al. (10), under the Creative Commons Attribution License 4.0 (https://creativecommons.org/licenses/by/4.0/).

Several visible features that may be identifiable on conventional MRI have been reported. On T2-weighted images, these include the presence of bilateral hyperintense rim lining the dorsolateral borders of the putamen (“putaminal rim” sign) and putaminal hypointensity in MSA-parkinsonian subtype (MSA-P) cases (11, 99). In addition, atrophy of the putamen, cerebellum, middle cerebellar peduncles (MCP), or pons may be noticed. In MSA-cerebellar subtype (MSA-C), visible features on T2-weighted and fluid-attenuated inversion recovery images may include cruciform pontine hyperintensity (“hot cross bun” sign; specificity 100%, sensitivity 58%; Figure 4a) and hyperintensity in the MCP (MCP sign; specificity 100%, sensitivity 50%; Figure 4b) (11, 99). Atrophy of the putamen, pons, cerebellum or MCP may be evident on T1-weighted images (Figure 4c). Notably, these signs have low sensitivity values and the appearance of these MRI markers can be influenced by image acquisition factors. For example, the “hot cross bun” sign was suggested to be more conspicuous on T2^*^-weighted vs. T2-weighted images (100), probably due to the presence of increased iron (100, 101).

The volume of the putamen was found to be significantly reduced in MSA vs. PD cases (102). While putaminal atrophy has been shown to differentiate MSA/MSA-P from PD with high specificity (~92%), a rather low sensitivity (~44%) was noted (99, 103). When putaminal changes are present and are asymmetric, they reliably correlate with the asymmetry of clinical features in patients with MSA-P. Several studies suggest that the analysis of multiple biomarkers may improve differentiation. For example, combined analysis of putaminal hypointensity visualized on gradient echo sequence along with putaminal atrophy improved the overall diagnostic accuracy of MSA-P cases vs. PD and PSP (104). Likewise, analyzing DTI and R2^*^ relaxation rate together enabled the identification of abnormal patterns unique to PD, PSP, and MSA disorders (61). On the other hand, a multimodal study (incorporating R2^*^, R1, and R2 mapping, magnetization transfer and DTI) found elevated putaminal R2^*^ values to be superior in the differentiation of MSA-P from PD, likely representing iron accumulation in the putamen (105).

In MSA vs. controls, volumetric and VBM studies commonly show striatonigral and olivopontocerebellar atrophy (11). Reduced volumes in the striatum and brainstem areas were observed in MSA vs. PD (28, 106), although with extensive overlap with PSP cases (28). In MSA-P, VBM studies also show atrophy in the primary motor and supplementary motor cortex indicating sensorimotor cortical degeneration (106, 107), as well as in the prefrontal and insular cortices compared to PD (106). Likewise, VBM analysis in MSA-P vs. PD revealed atrophy in the superior cerebellar peduncle (SCP), MCP, cerebellum, pons, midbrain, and putamen, but not in the globus pallidus (71). Cerebellar atrophy was evident in both MSA-P and MSA-C subtypes to varying degrees (28, 108). In an analysis of cerebellar neuroanatomical differences in MSA subtypes, a relatively greater GM atrophy was observed in MSA-C cases (vs. MSA-P) in the right Crus II—a cerebellar region involved in executive motor control (108). Both MSA-P and MSA-C patients may exhibit putaminal and infratentorial atrophy with considerable overlap, however, putaminal/supratentorial atrophy was more predominant in MSA-P subtype, whereas infratentorial atrophy was more prominent in MSA-C subtype (109, 110).

MSA patients with cognitive impairment showed volumetric reduction in the left dorsolateral prefrontal cortex vs. cognitively-normal MSA cases (111). The authors suggested that cortical pathology contributes minimally to cognitive deficits in MSA, whereas frontostriatal degeneration may be the primary driver of cognitive dysfunction (as per the concept of “subcortical cognitive impairment”) (111). Another study identified cortical thinning in the parahippocampal and lingual cortices in MSA with dementia vs. cognitively-normal MSA patients (112).

#### Diffusion-Weighted and Diffusion Tensor Imaging in MSA

In MSA-P, higher *D* was identified in the putamen compared to subjects with PD, MSA-C and controls (113). Similarly, reduced FA and elevated apparent diffusion coefficient (ADC) values were observed in MSA-P in the putamen, cerebellum and pons vs. PD and controls (114). Combined analysis of elevated T2^*^ relaxation rate and putaminal *D* allowed discrimination of PD from MSA-P with high accuracy (113). Likewise, a multiregional evaluation of diffusivity changes in the pons, putamen and cerebellum was found to be more useful than single-region analysis (114). Compared to PSP, increased *D* was observed in MCP and pons in MSA cases, which correlated with cerebellar ataxia in these regions (115). In a comparison between MSA-P and MSA-C subtypes, elevated ADC values were observed in the putamen and pons in MSA-P cases vs. MSA-C, and in the cerebellum and MCP in MSA-C cases vs. MSA-P, highlighting distinct microstructural damage in these subtypes (116). Microstructural changes in the WM may be more pronounced early in the disease in MSA-C than in MSA-P (110). In MSA patients with cognitive impairment, a greater involvement of the cerebrum (specifically, reduced FA in the anterior corpus callosum) was identified compared to cognitively-normal MSA patients (117).

Using the bi-tensor diffusion analysis model, free-water was found to be elevated in the posterior SN in PSP, MSA, and PD vs. controls, however, this increase was significantly greater in PSP than in PD/MSA patients (118). In addition, depending upon the disease severity, free-water may be elevated in both the anterior and posterior SN in PD, MSA and PSP vs. controls (66). Free-water-corrected FA was elevated in MSA in the putamen and caudate vs. controls; whereas, it was decreased in the thalamus and increased in the SCP in MSA vs. PSP (66). Using a machine learning algorithm, combined analysis of free-water and free-water-corrected FA derived from selective regions-of-interest achieved excellent separation among PD, MSA, and PSP cases (66). Unsupervised machine learning-based classification of PD, MSA-P and MSA-C patients using multimodal neuroimaging measures (GM density, T2^*^ relaxation rate, and DTI) have also been pursued with favorable results (119). Free-water imaging using advanced diffusion models may become an effective tool in the differential diagnosis of parkinsonian disorders in the future.

#### Proton Magnetic Resonance Spectroscopy in MSA

Compared to controls, NAA/Cr ratios were smaller in the putamen in MSA-P, and in the pontine base in both MSA-P and MSA-C cases (120). Lower NAA/Cr ratios in the putamen and pontine base best discriminated MSA-P cases from PD (120). In another study, no significant differences were observed in the metabolites examined between MSA-P and PD, suggesting similar metabolic alterations in the two disorders (71). Likewise, cerebellar NAA/Cr and NAA/myo-inositol levels in MSA-P subjects were similar to those seen in PD (77). In MSA-C patients, cerebellar NAA/Cr and NAA/myo-inositol ratios were significantly reduced compared to PD, MSA-P, PSP-Richardson's syndrome (PSP-RS), and controls, whereas cerebellar myo-inositol/Cr ratios were elevated in MSA-C compared to controls (77).

### Progressive Supranuclear Palsy (PSP)

#### Magnetic Resonance Imaging in PSP

**Figure 5:**
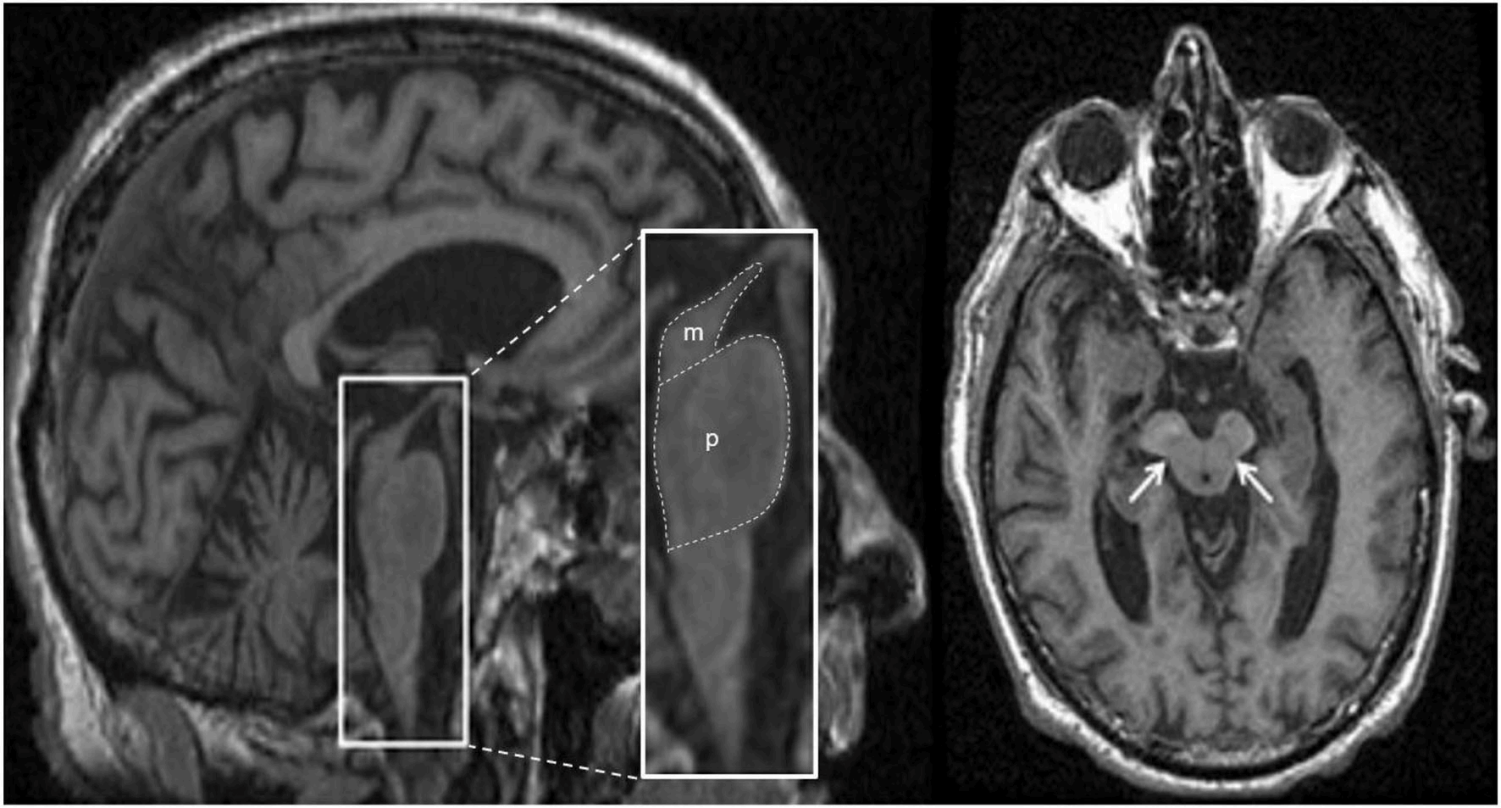
Magnetic resonance imaging of a patient clinically-diagnosed with progressive supranuclear palsy. The left image is a sagittal T1-weighted sequence showing the “hummingbird” sign (smaller box), while the right image is an axial T1-weighted sequence showing the “morning glory” sign (arrows); both features are seen in progressive supranuclear palsy. The pons (p) and midbrain (m) areas are also shown (larger box), and their ratios have been used to calculate an index to assist in the diagnosis (128). Figure adapted from Saeed et al. (10), under the Creative Commons Attribution License 4.0 (https://creativecommons.org/licenses/by/4.0/).

Structural MRI typically shows atrophy of the midbrain and SCP in PSP compared to PD, MSA-P, CBD/CBS and controls (121–124). Several morphological markers suggestive of PSP on MRI have been reported, including midbrain atrophy compared to pons (“hummingbird” sign; specificity ~99.5%, sensitivity ~51%; Figure 5), atrophy of the midbrain tegmentum (“morning glory” sign, specificity ~97%, sensitivity ~37%; visualized as concavity of the lateral margins of the midbrain tegmentum on axial images; Figure 5) (125–127), midbrain T2 hyperintensity, as well as atrophy of the midbrain tegmentum with relative preservation of the midbrain tectum and cerebral peduncles (“mickey mouse” sign, visualized as rounded rather than rectangular midbrain peduncles on axial images) (99, 125). Importantly, the “hummingbird” and “morning glory” signs had high specificity but low sensitivity (99). Furthermore, image acquisition parameters may influence the appearance of these morphological features (4).

In PSP vs. controls, reduced volumes were reported in the brainstem, midbrain, and frontal GM (129), however, only the midbrain volume showed minimal overlap with the normal range on a case by case basis (129). In PSP-RS, atrophy of the midbrain was the predominant feature (4, 130). In a pathology-proven sample, atrophy of the midbrain and SCP was associated with PSP, whereas frontoparietal and pallidum degeneration in the absence of significant brainstem atrophy was suggestive of CBD (123). The premotor cortices and supplementary motor area were involved in both PSP and CBD cases (123). Besides midbrain atrophy, VBM studies also show degeneration in the subcortical structures including pons, thalamus and striatum, as well as widespread cortical atrophy in the frontal, prefrontal, insular, premotor and supplementary motor areas vs. controls (121, 131, 132). Moreover, WM degeneration was reported in the pulvinar, dorsomedial and anterior nuclei of the thalamus, superior and inferior colliculi, as well as in the mesencephalic, and frontotemporal regions (131, 132). Pontine atrophy rates were significantly slower in PSP vs. MSA-P, and rates in the frontal lobe and midbrain in PSP were associated with executive and motor impairment, respectively (133). The midbrain atrophy rate may serve as an effective outcome measure in PSP clinical trials (134). In addition, the support vector machine classification method yielded accuracy rates >80% for predicting PSP diagnosis using disease-specific regions-of-interest (pallidum, putamen, caudate nucleus, thalamus, midbrain and insula) compared to the whole-brain approach (135).

Predominant midbrain atrophy has been incorporated as a supportive imaging feature in the Movement Disorders Society's PSP diagnostic criteria (3). The ratios of the pons to midbrain area (P/M) (as shown in Figure 5) and MCP to SCP widths (MCP/SCP) were found to be larger in PSP compared to PD, MSA-P and controls (specificity and sensitivity, 100%) (128, 136). Using these ratios, an index was calculated as [(P/M) x (MCP/SCP)], termed the “magnetic resonance parkinsonism index,” which proved to be highly sensitive and specific for distinguishing PSP from PD, MSA-P and controls (128). A revised version incorporating the width of the third ventricle has been proposed [“magnetic resonance parkinsonism index” × (third ventricle width)/(frontal horns width)], which showed superior sensitivity (100%) and specificity (94.3%) in differentiating PSP-parkinsonism patients with slowness of vertical saccades from PD (137). It is important to recognize that midbrain atrophy may not be evident in all PSP subtypes and thus, atrophy patterns from other brain regions (such as the globus pallidus, frontal lobe, and cerebral peduncle) may provide additional information (138). It is unknown whether the above quantitative measures can distinguish PSP patients at early disease stages, or whether PSP subtypes with less pronounced midbrain atrophy can be distinguished, which provides impetus for further research.

#### Diffusion-Weighted and Diffusion Tensor Imaging in PSP

Compared to controls, diffusion imaging studies in PSP show variable findings and may reveal the following: increased *D* or decreased FA in the decussation of SCP, orbitofrontal WM, thalamus, cingulum, motor and supplementary motor area, as well as in the inferior fronto-occipital fasciculus, superior longitudinal fasciculus, anterior corpus callosum, arcuate fasciculus, posterior thalamic radiations, and internal capsule (115, 132, 139, 140). Elevated ADC values were observed in the putamen in PSP vs. controls over a 2 year period (141). Increased *D* in the decussation of SCP may discriminate PSP from MSA and PD (115), whereas increased ADC values in the putamen, globus pallidus and caudate nucleus may help distinguish PSP cases from PD (142). Another study calculated the FA score per subject for regions hypothesized to be involved in PSP (i.e., the SCP and frontal WM region), and reported >85% sensitivity and specificity for differentiating PSP from PD/DLB cases (143).

A multimodal study integrated volumetric MRI, DTI and neuromelanin-sensitive imaging, and identified several predictors for separating PSP-RS from controls (144). The best predictor was the neuromelanin-based SN volume followed by FA in the midbrain (144). The separation of PSP-RS cases from PD was achieved using neuromelanin-based SN volume, pons FA values, midbrain and globus pallidus volumes, and basal forebrain FA values (144). Another study identified greater atrophy, decreased FA, and increased *D* in the SCPs bilaterally in PSP-RS patients as compared to PD and controls (145). More advanced neuroimaging techniques have identified changes in the free water in the posterior SN (118). Specifically, free-water values derived from the bi-tensor diffusion model were significantly elevated in PSP in the posterior SN vs. MSA, PD and controls, and were observed in a characteristic pattern: PSP > MSA/PD > HC (118). In addition, free-water-corrected FA was elevated in PSP in the caudate, putamen, thalamus and vermis with accompanying decreases seen in the SCP and corpus callosum compared to controls (66).

Other studies have compared diffusion imaging parameters in PSP subtypes. Lower FA values were exclusively detected in the SCP in PSP-RS vs. PSP-parkinsonism patients, implicating SCP's involvement in postural instability (146). PSP patients with vertical supranuclear gaze palsy exhibited lower FA values in the midbrain vs. those with slowness of vertical saccades, highlighting the role of midbrain atrophy in vertical ocular dysfunction (146). PSP-RS patients also showed more severe and widespread diffusion abnormalities vs. PD, which reflects greater microstructural damage as consistent with greater overall brain atrophy often found in PSP-RS than in PD cases (147). Moreover, damage to the SCPs may be detected in both PSP-RS and PSP-parkinsonism subtypes (130, 145). When the two subtypes were directly compared, PSP-RS patients showed decreased FA and increased *D* in the left SCP vs. PSP-parkinsonism patients (145). Computer-aided diagnosis of PSP and its subtypes may be possible using diffusion-weighted/DTI measures (145, 147).

#### Proton Magnetic Resonance Spectroscopy in PSP

In PSP, reductions in the NAA/Cr ratios in the LN, brainstem, centrum semiovale, frontal, and precentral cortex, as well as reductions in the NAA/choline values in the LN are observed relative to controls (148, 149). A more prominent decline in NAA/Cr ratio was noted in the putamen vs. PD and MSA (150). PSP-RS patients had reduced cerebellar NAA/Cr and NAA/myo-inositol ratios vs. controls, and reduced cerebellar NAA/Cr ratio vs. PD patients (77). Compared to controls, PSP patients also showed a decrease in scyllo-inositol concentration (a stereoisomer of inositol) and scyllo-inositol/Cr ratio in the supplementary motor area, and both of these metabolic measures were directly related to attention and working memory functions (151). The pathological significance of scyllo-inositol reduction in PSP is currently uncertain (151).

### Corticobasal Degeneration/Syndrome (CBD/CBS)

#### Magnetic Resonance Imaging in CBD/CBS

**Figure 6:**
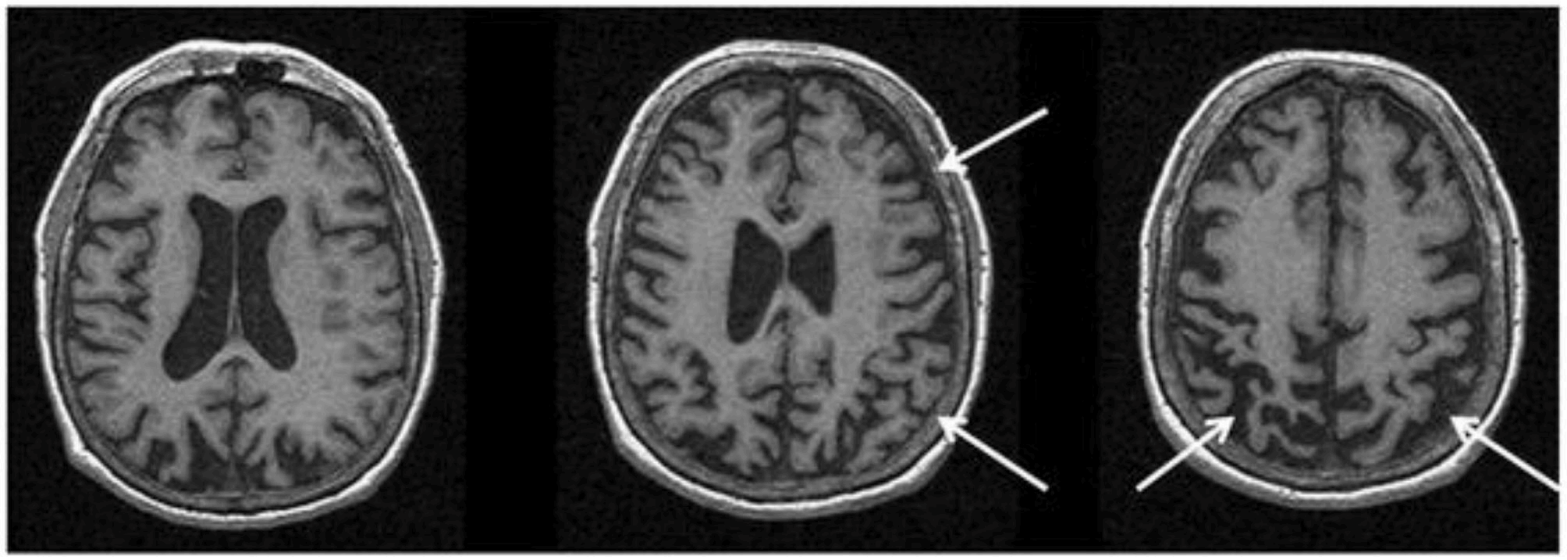
Magnetic resonance imaging of a patient with a pathology-proven diagnosis of corticobasal degeneration. Serial axial T1-weighted sequences are presented showing right greater than left parietofrontal atrophy commonly seen in corticobasal syndrome. Figure reproduced from Saeed et al. (10), under the Creative Commons Attribution License 4.0 (https://creativecommons.org/licenses/by/4.0/).

In CBS/CBD, asymmetrical cortical atrophy in the frontoparietal lobe is commonly observed (Figure 6), contralateral to the clinically more affected side of the body (however, laterality may not be present in all cases). In CBS vs. controls, an asymmetric pattern of atrophy in the bilateral premotor cortex, superior parietal lobules, and striatum was identified (121). Compared to PSP, greater atrophy was observed in the dorsofrontal and parietal cortices in CBS (121, 129), whereas midbrain atrophy was more pronounced in PSP vs. CBS (121). Likewise, greater asymmetric GM degeneration in the inferior frontal and premotor cortex, parietal operculum, superior temporal gyrus, and hippocampus was detected, along with decline in FA primarily in the frontoparietal region vs. controls (152). In a meta-analysis of VBM studies, although a significant overlap was detected among PSP, MSA-P, and CBS cases, more prominent atrophy in the superior parietal lobe was observed in CBS (153). Importantly, CBS can be associated with significant pathological heterogeneity difficult to predict based on clinical presentation in life (15, 154). Thus, neuroimaging patterns using pathology-proven samples can improve diagnostic accuracy.

On MRI, the pattern of atrophy in CBS aligns with the “true” underlying pathology. Although, GM atrophy in a clinically-diagnosed CBS group was observed in the premotor cortices, supplemental motor area and insula, the pattern of atrophy aligned more closely with neuropathological diagnosis (155). For example, frontotemporal atrophy in CBS was associated with frontotemporal lobar degeneration with transactive response DNA binding protein-43 kDa (TDP-43) pathology, whereas temporoparietal atrophy was related to AD pathology (155). In contrast, focal atrophy predominantly involving the premotor cortex and supplementary motor area was observed in those pathologically diagnosed with CBD and PSP, although more severe changes in these regions suggested CBD over PSP (155). The degree of gross global atrophy is typically more severe in CBD vs. PSP (156). Another pathology-confirmed study observed GM degeneration in the dorsal prefrontal and perirolandic cortex, striatum, and brainstem in CBD vs. controls (6). Furthermore, in CBS due to frontotemporal lobar degeneration (tau or TDP-43), atrophy pattern progressed into the prefrontal cortex, striatum and brainstem, while in CBS due to AD, atrophy stretched into the temporoparietal cortex and precuneus regions (6). The predominant clinical syndrome in CBS (extrapyramidal vs. cognitive) was associated closely to the regional atrophy patterns (123). On fluid-attenuated inversion recovery images, subcortical WM hyperintensities with ventricular dilation (greater in the more affected lobe) were reported (157), however, these changes are not specific to CBS/CBD.

#### Diffusion-Weighted and Diffusion Tensor Imaging in CBD/CBS

Elevated *D* and reduced FA were detected in the posterior truncus of the corpus callosum in CBS compared to PD and controls suggesting transcallosal fiber degeneration (59). In addition to the corpus callosum (59, 152, 158), decreased FA in CBS was observed in the long frontoparietal connecting tracts, intraparietal associative fibers, and sensorimotor cortical projections (152). Similarly, reduced FA and increased *D* were noted in the WM of premotor, prefrontal and motor cortices, as well as in the middle cingulate bundle vs. controls—changes that were more pronounced contralateral to the more affected side (158). Compared to PSP, a more asymmetric, supratentorial and posterior pattern of WM tract degeneration was reported in CBS with greater involvement of the splenium of the corpus callosum, WM of the motor and premotor cortices, as well as the parietal lobes (158).

#### Proton Magnetic Resonance Spectroscopy in CBD/CBS

NAA/Choline and NAA/Cr levels were reduced in CBS patients in the frontoparietal cortex, LN and centrum semiovale vs. controls (149, 159). Lower NAA/Choline levels in the frontoparietal lobe may help differentiate CBS cases from PSP (149). In the parietal cortex of CBS patients, the NAA/Choline values were reduced contralateral to the clinically more affected side (159). A more pronounced reduction in NAA/Cr values was observed in the frontal cortex and putamen vs. PD, MSA and vascular parkinsonism, with prominent asymmetry in NAA/Cr ratios in the putamen (150). Lower putaminal NAA levels have been reported across the PD, MSA, PSP and CBS patients to varying degrees. Thus, laterality of metabolite ratios observed in the putamen in CBS may be helpful in differentiation in some cases.

## Functional Magnetic Resonance Imaging in Parkinsonian Disorders

Using resting-state and task-based functional MRI, several large-scale networks involved in motor, cognitive, and affective processes have been identified (Table 3). Impairments in these networks as well as in specific circuits (e.g., basal ganglia thalamocortical circuit and cortical-subcortical sensorimotor circuit) have been associated with motor and non-motor symptoms in PD and atypical PS. Reduced resting-state functional connectivity between the striatum and the thalamus, midbrain, pons and cerebellum was observed in PD, highlighting connectivity alterations within the brainstem (160). Within the striatum, a greater change in connectivity was evident in the posterior putamen, followed by the anterior putamen and caudate (160) as consistent with the patterns of striatal dopaminergic dysfunction in PD (161–163). Connectivity changes between the striatum and sensorimotor and visual cortical areas as well as the supramarginal gyrus were also evident (160) probably highlighting dysfunction of the cortical-subcortical sensorimotor circuit in PD (164). Furthermore, reduced resting-state functional connectivity within the basal ganglia network allowed differentiation of PD cases in the drug-off state from controls (sensitivity 100%, specificity 89.5%) (165). Conversely, increased functional connectivity was detected in associative and limbic connections in PD likely indicating compensatory changes due to dopaminergic deficits and the ensuing alterations in related circuits (164).

**Table 3:** An overview of 4 core brain networks.

**Networks^‡^**	**Description**	**Common network-associated regions**
Default mode network	Involved in endogenously mediated activities at rest including self-referential and social cognitive processes, and it is inactive during external goal-oriented processes	Posterior cingulate cortex, medial prefrontal cortex, precuneus, and inferior parietal and medial temporal cortices
Salience network	Involved in the bottom-up detection of salient stimuli that require dynamic switching between the central executive and default-mode networks, in order to keep cognitive resources focused on task-relevant goals	Anterior cingulate and anterior insular cortices, as well as amygdala, thalamus, hypothalamus, ventral striatum, and substantia nigra
Central executive network	Involved in external goal-oriented and cognitively demanding processes including working memory, planning, and decision making	Dorsolateral prefrontal cortex and posterior parietal cortex
Sensorimotor network	Involved in the execution of voluntary motor activities	Primary motor cortex, supplementary motor area, primary and secondary sensory cortices

*^†^Several other networks, including frontoparietal network, dorsal and ventral attentional networks, and visual network (among others) have also been postulated*.

In tremor-dominant PD, the globus pallidus internus and putamen exhibited elevated functional connectivity with the cerebellothalamic circuit that modulates tremor amplitude. It was suggested that basal ganglia degeneration (and the resulting dopamine deficiency) can cause tremors by disrupting cerebellothalamic circuit signaling (166). PD patients with freezing of gait showed abnormal functional connectivity in the pedunculopontine nucleus, which primarily affected the corticopontine-cerebellar pathways and visual temporal areas involved in visual processing (167). These findings are consistent with observations seen in DTI structural connectivity studies (168). Furthermore, PD patients with sleep disturbance showed changes in cortical functional connectivity within the default mode network, central executive network, and dorsal attention network vs. PD patients without sleep disturbance (169). Hallucinations in PD were found to be associated with functional connectivity changes within the default mode network and visual processing areas implicating networks involved in perceptual and attentional processing (170).

Compared to PD, patients with MSA displayed reduced cerebellar connectivity within multiple brain networks as well as the striatum (171). Overlap in functional connectivity was noted in PD and PSP patients within the thalamus, striatum, and prefrontal cortex, however, not surprisingly, the PSP group showed more extensive functional connectivity disruptions throughout the brain (particularly in the midbrain, precentral gyrus, parietal cortex, basal ganglia, and cerebellum) (172). Another study identified deficits in the resting-state functional connectivity in PSP cases in the rostral midbrain tegmentum network (173). In CBS, decreased functional connectivity was reported in the right central operculum, middle temporal gyrus, and posterior insula, whereas an increase in connectivity was identified in the anterior cingulum, medial superior frontal gyrus, and bilateral caudate nuclei (174). Thalamic functional connectivity was decreased in both PSP and CBS groups in multiple cortical, subcortical, and cerebellar regions (175). In contrast, whole brain functional connectivity of the dentate nucleus differed between PSP and CBS: it was reduced in the subcortical and prefrontal cortical areas in PSP, whereas it increased asymmetrically in the frontal cortex in CBS (175).

In a longitudinal task-based functional MRI study that incorporated a motor control paradigm, a decline in activity within the putamen and primary motor cortex was identified over 1 year in PD patients vs. controls (176). Conversely, a more widespread and unique pattern of functional changes were observed in MSA and PSP patients compared to PD. In MSA, changes were exclusively extrastriatal (i.e., the primary motor cortex, supplementary motor area and superior cerebellum) (176). In PSP, all regions-of-interest were less active at 1 year compared to baseline, including the contralateral putamen, ipsilateral putamen, contralateral primary motor cortex, contralateral supplementary motor area, and ipsilateral superior cerebellum (176).

Changes in functional connectivity after symptomatic treatment interventions have also been observed. For example, increase in functional connectivity was identified in the supplementary motor area (part of the sensorimotor resting-state network) after levodopa administration in drug-naïve PD patients (177). The sensorimotor system was suggested to be one of the targets of acute levodopa treatment (177). Likewise, the administration of dopaminergic medication resulted in enhanced connectivity within the basal ganglia network (165). Finally, repetitive transcranial magnetic stimulation can improve motor symptomatology by influencing functional hubs connecting to motor-related networks, including the default mode, cerebellar, and limbic networks (178).

## Transcranial B-Mode Sonographic Imaging in Parkinsonian Disorders

### Echogenicity in Substantia Nigra

**Figure 7:**
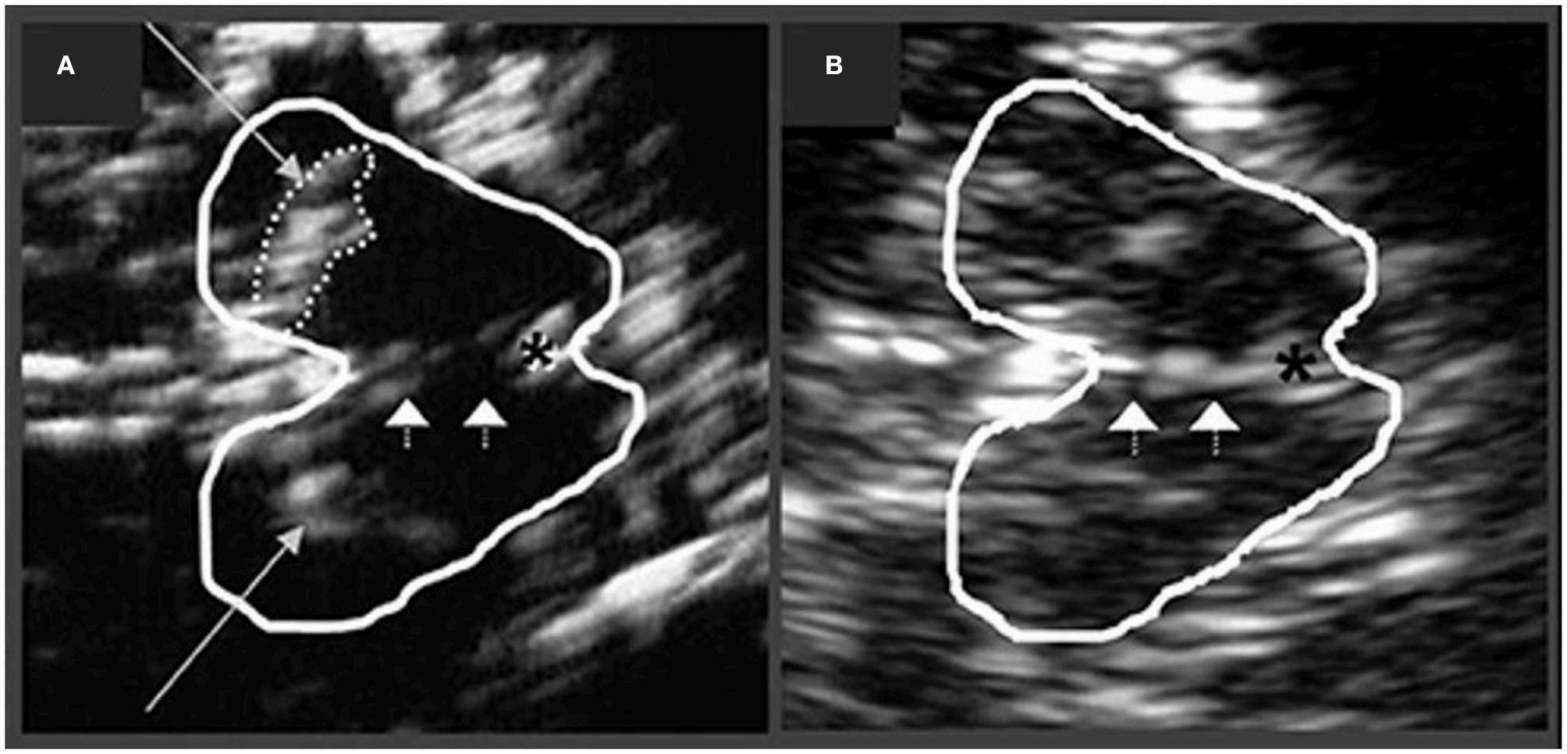
Transcranial sonographic image outlining the butterfly-shaped midbrain at the mesencephalic plane. In **(A)**, enlarged area of echogenicity at the anatomical site of substantia nigra (long arrows) is depicted, as may be seen in Parkinson's disease patients. In addition, interrupted echogenic line of the raphe can be observed (short arrows). In **(B)**, normal midbrain echogenicity is shown. The aqueduct is indicated by an asterisk. Figure adapted from Richter et al. (182), under the Creative Commons Attribution License (https://creativecommons.org/licenses/by/4.0/).

In PD, increased echogenicity of the SN is commonly observed, which can be visualized at the mesencephalic plane as an enlarged, lighter (i.e., mildly echogenic) region within the darker mesencephalon (179–182) (Figure 7). Similar to idiopathic PD, increased SN echogenicity is seen in PD patients carrying *LRRK2* and *GBA* mutations (183). Although, the precise etiology of SN hyperechogenicity is under research, it is likely due to the known nigral pathology and associated accumulation of free (unbound) iron within the SN.

SN hyperechogenicity may be present in preclinical stages as detected in those with increased risk of PD, e.g., individuals with a family history of PD (184). The area of SN hyperechogenicity remained stable in PD over a 5 year period (185) suggesting that this feature may be considered an early “trait” marker of vulnerability, as opposed to a marker of progression in PD. Indeed, studies have not consistently shown a correlation between SN hyperechogenicity and disease severity or duration (181, 186–188). Likewise, no correlation has been observed between SN hyperechogenicity and the degree of presynaptic DAT loss in PD (189). SN hyperechogenicity may not differ based on PD laterality and has been observed in both hemispheres, irrespective of the clinically affected side (187).

It is also important to note that ~10% of healthy controls, as well as ~16% of patients with essential tremor may show elevated echogenicity in the SN (190), thus limiting the utility of this “echofeature” as a standalone biomarker of PD. For example, in a 3 year longitudinal study, baseline SN hyperechogenicity was evident not only in PD, but also in patients with essential tremor, who subsequently developed parkinsonian symptoms during the follow-up period (191).

Increased echogenicity of the SN, particularly when marked, has been reported to differentiate PD cases from atypical PS (PSP and MSA as a group) with good sensitivity (~91%) and specificity (~82–96%) (179, 192). When experienced examiners are available, this technique can be resourceful in differentiating PD from atypical PS. However, patients with DLB and PDD also present with hyperechogenic SN in frequencies similar to those in PD. For example, ~80% of DLB cases showed hyperechogenic SN bilaterally, and thus the differential diagnosis in PDD/DLB relies upon other clinical features and diagnostic biomarkers (193). Nevertheless, it is suggested that the discrimination between DLB and PDD using transcranial sonography may be made by combining SN echogenic sizes, asymmetry indices, and onset age (sensitivity 96%, specificity 80%) (193). Hyperechogenic SN can also be seen in CBS (~60–80%) and PSP disorders [~10–20%, especially in PSP-parkinsonism subtype (194)]; however, studies in CBS and PSP are limited by small sample sizes and lack of neuropathological confirmation (195–197).

### Echogenicity of the Lentiform Nucleus

Increased echogenicity of the LN is a feature noted in some atypical PS and may be of diagnostic utility when examined together with SN echogenicity. In healthy individuals, the LN is visualized as an isoechogenic structure, located between the caudate nucleus and thalamus. Hyperechogenic LN may support a diagnosis of atypical PS when seen together with normoechogenic SN (188). Specifically, this echogenic profile was observed in MSA-P and PSP patients with good sensitivity (100%), albeit with a low specificity (~59%). Normal echogenicity of the SN alone aligned with a diagnosis of MSA-P (sensitivity 90%, specificity 98%), while increased echogenicity of the LN alone was unhelpful in the differential diagnosis of parkinsonian syndromes since PD and PSP patients may show this feature to some degree (197). Enlargement of the third ventricle can also be examined using transcranial sonography. Ventricular enlargement >10 mm together with LN hyperechogenicity may indicate PSP (197), whereas normal LN echogenicity was observed in CBS patients (195). Given the considerable heterogeneity evident in atypical PS, more studies are needed to develop reliable sonographic profiles of these disorders.

## Spect and Pet Imaging of the Dopaminergic System in Parkinsonian Disorders

### Presynaptic Dopamine Transporter (DAT)

Normal DAT binding on ^123^I-FP-CIT SPECT can be visualized as two bright symmetric “comma-shaped” regions, signifying intense activity in the striatum (striatum includes caudate nucleus and putamen; Figure 2). Any change in this activity on ^123^I-FP-CIT SPECT may indicate presynaptic nigrostriatal injury (198, 199). Overall, DAT SPECT shows normal presynaptic nigrostriatal activity in normal individuals, essential tremor cases, and in drug-induced or psychogenic/functional parkinsonism (200–202).

Conversely, reduced DAT binding on SPECT has been observed in PD, DLB/PDD, MSA, and PSP patients to varying degrees suggesting nigrostriatal degeneration (200, 203–205). The utility of DAT SPECT in differentiating PD in early stages from normal subjects and cases with essential tremor and vascular parkinsonism with high accuracy has been suggested (206). In PD, the posterior putamen exhibits earlier and more severe reduction in activity compared to the anterior putamen or caudate nucleus (161). The decline in DAT signal is often more pronounced in the hemisphere contralateral to parkinsonian symptomatology, whereas binding may appear symmetric in cases with symmetric motor deficits (161). Striatal DAT SPECT binding has been shown to correlate with PD severity and motor impairment (202, 207–209). Reduced DAT binding can differentiate patients with PD and DLB from AD (210, 211). In a recent population-based study, striatal DAT deficits (particularly in the caudate nucleus) were associated with shorter survival in PD patients (212).

Similar to SPECT, ^18^F-dopa PET studies evaluating the presynaptic nigrostriatal dopaminergic system have found reduced radiotracer uptake in PD, MSA-P, PSP and DLB groups vs. controls, whereas normal uptake was observed in cases with essential tremor (205, 213). Specifically, ^18^F-dopa PET measures the activity of aromatic amino acid decarboxylase (AADC) enzyme, which converts ^18^F-dopa into ^18^F-dopamine, providing an approximation of dopaminergic storage levels. In PD, decreased ^18^F-dopa uptake was first evident in the posterior putamen, followed by anterior putamen and caudate nucleus, contralateral to the clinically affected side (162, 163). Striatal and putaminal ^18^F-dopa uptake have been shown to associate with PD progression and motor severity, respectively (163). Other PET radiotracers, such as ^11^C-DTBZ, can be used to evaluate the presynaptic monoaminergic system by labeling the vesicular monoamine transporter type 2 (VMAT2)—a presynaptic transmembrane protein essential for packaging and storing monoamines (including dopamine) in synaptic vesicles. Reduced VMAT2 binding in the striatum was detected in PD on ^11^C-DTBZ PET (214). As identified in ^18^F-dopa PET, the greatest regional decrease in VMAT2 binding in PD (using ^18^F-AV-133) was observed in the posterior putamen, followed by the anterior putamen and caudate nucleus (215). Notably, due to ongoing compensatory changes in response to neurodegeneration (AADC upregulation, presynaptic DAT down-regulation), the nigrostriatal presynaptic dopaminergic injury may be underestimated using PET and SPECT imaging (214). Furthermore, an approximated 10–20% of clinically diagnosed PD cases that were enrolled in neuroprotective trials of PD and underwent DAT imaging presented with “scans without evidence of dopaminergic deficit” (216). Studies have shown this group of cases to be quite heterogeneous and [among other reasons (10)] represent a clinical misdiagnosis of PD in most cases (10, 216). Reduced DAT binding using ^11^C-methylphenidate PET has also been identified in clinically-unaffected mutation carriers of *LRRK2* gene associated with dominant inheritance of PD (217). Elevated dopamine turnover in the putamen was suggested to be an even more sensitive subclinical indicator of PD in *LRRK2* mutation carriers compared with reduced dopaminergic terminal integrity as assessed by VMAT2 and DAT binding (218).

DAT SPECT imaging can be invaluable in differentiating DLB from other forms of dementia (219). For example, an abnormal DAT scan can enhance the diagnostic certainty of DLB from “possible” to “probable” (220), and assist in differentiating DLB without clinically significant parkinsonism from AD (221–223). The development of parkinsonism over 6 months was associated with abnormal baseline ^123^I-FP-CIT SPECT scan in possible DLB patients (224). Although, an abnormal DAT scan supports the diagnosis of DLB, a normal scan does not exclude DLB altogether, including those cases that present with minimal brainstem involvement (223).

In MSA-P, signal loss on DAT SPECT was greater over time in the caudate and anterior putamen vs. PD, as consistent with a relatively faster rate of disease progression in MSA-P (225). Patients with CBS may show striatal DAT SPECT reduction with greater hemispheric asymmetry vs. PD (226, 227). Furthermore, patients with CBS/CBD may show normal nigrostriatal DAT SPECT scans, especially early in the course of the disease, suggesting that nigrostriatal degeneration may be a late pathological feature of CBD (228). Patients with PSP tend to exhibit more pronounced but fairly uniform DAT loss in the striatum (204). In contrast to PD, a relatively uniform involvement of presynaptic striatal dopaminergic neurons was observed in PSP, as evidenced by lower striatal-to-occipital but higher putamen-to-caudate DAT binding ratios (229, 230). Likewise, a more symmetric pattern of DAT loss was detected in PSP vs. PD and MSA-P (202, 229, 230), with the index of asymmetry significantly greater in PD relative to PSP (230). A recent meta-analysis further confirmed reduced DAT activity in the caudate nucleus and putamen in PSP vs. PD and MSA-P, and in MSA-P vs. MSA-C (231). Investigations using ^18^F-dopa PET mirror these SPECT-based findings as follows: putaminal uptake was significantly lower in atypical PS and PD vs. controls (232–234). However, a more severe decline was noted in the caudate head in atypical PS vs. PD (234). As compared to PD, putamen and caudate regions were equally abnormal in PSP (232). Despite these findings, the presynaptic striatal binding patterns using PET and SPECT are currently unreliable in differentiating parkinsonian disorders on a case-by-case basis. Finally, using ^99m^Tc-TRODAT-1 SPECT, lower putaminal DAT uptake was associated with shorter time of conversion from idiopathic RBD diagnosis to an α-synucleinopathy vs. those with higher putaminal DAT uptake (235). This may suggest a predictive role of nigrostriatal damage in idiopathic RBD in terms of conversion to an α-synucleinopathy (235). However, an abnormal DAT scan was found to be less sensitive than motor features in predicting phenoconversion from idiopathic RBD to overt neurodegenerative syndrome (of PD, LBSD, and MSA) (236).

### Postsynaptic Dopamine D2 Receptor

In drug-naïve PD patients compared to controls, binding potential for the G-protein-coupled dopamine D2 receptors measured using ^11^C-raclopride PET may appear normal or upregulated contralateral to the clinically affected side (237–239). Similarly, striatal dopamine D2 receptor upregulation was observed in drug-naïve PD patients using SPECT ligands (^123^I-IBZM and ^123^I-IBF) probably suggesting compensatory changes secondary to nigrostriatal denervation, with higher upregulation detected in the posterior putamen (161, 240). In medicated PD cases, postsynaptic D2 receptor binding was reduced or within the normal range compared to controls in PET and SPECT studies (161, 200, 237, 241). Normal D2 binding potential was also observed in patients with DLB and essential tremor (161, 200), while reductions were reported in atypical PS cases (239).

In PSP vs. controls, reduced D2 receptor binding was detected in PET and SPECT studies (200, 239, 242). Likewise, D2 binding reductions were noted in MSA patients compared to PD (233, 241) and controls (200, 233, 241, 242) correlating with striatal glucose hypometabolism (241). In CBS, studies typically show preservation of postsynaptic D2 receptors, although inconsistently, which is not surprising given the pathologic heterogeneity evident in this disorder (200, 203, 243).

The posterior putamen to caudate binding ratios were >1 in almost all drug-naïve and medicated PD and PSP cases (161). In contrast, this ratio was <1 in most MSA cases indicating greater loss of D2 receptors in the posterior putamen in MSA (161). This finding is consistent with a ^11^C-raclopride PET study, whereby elevated caudate-to-putamen and anterior-to-posterior putamen D2 receptor binding ratios were observed in MSA-P vs. PD suggesting greater D2 receptor loss in the posterior putamen in MSA-P cases (244).

Combining SPECT-based presynaptic DAT and postsynaptic D2 receptor imaging may improve diagnostic capacity (245). For example, a study suggested increased accuracy in differentiating PD from atypical PS using a multidimensional combination of striatal presynaptic DAT imaging, postsynaptic D2 receptor imaging, and myocardial scintigraphy [evaluates cardiac postganglionic sympathetic fiber function, which is significantly impaired in early PD and LBSD, as reviewed in (10)] (246). Further studies with pathology proven samples are needed to improve the utility of postsynaptic D2 receptor imaging in differentiating parkinsonian disorders.

## Spect Imaging of Cerebral Perfusion in Parkinsonian Disorders

Cerebral perfusion SPECT evaluates the metabolic status of brain tissue by quantifying changes in the regional cerebral blood flow using various radiotracers (Table 2). Occipital hypoperfusion is frequently observed in DLB, however, it may not be present in all cases on an individual basis. When present, it should raise the possibility of DLB as the underlying cause of the disease. Using ^99m^Tc-HMPAO as a radiotracer, temporoparietal hypoperfusion was detected in both AD and DLB cases to varying degrees vs. controls, whereas occipital hypoperfusion was the differentiating feature in DLB vs. AD (247). Occipital hypoperfusion has also been detected using other SPECT radiotracers (e.g., ^123^I-IMP and ^99m^Tc-ECD). Perfusion SPECT was unable to differentiate PDD cases from DLB, revealing similar perfusion profiles in some studies (248, 249). In addition, hypoperfusion in the left occipital region along with worse episodic memory performance was found to distinguish DLB patients without visual hallucinations from CBS at earlier disease stages (250).

Patients with CBS tend to show asymmetric perfusion profiles (251, 252), however, asymmetry may not be observed in all cases. Compared to PSP (using ^123^I-IMP tracer), asymmetrically reduced perfusion was noted in CBS in the inferior prefrontal, sensorimotor, and posterior parietal cortices, with overlap in the medial frontal region (252). Perfusion asymmetry may serve as a supportive feature when differentiating CBS from other atypical PS patients. The differentiation of LBSD from atypical PS may be feasible using an automated image-based classification system, which incorporates striatal DAT uptake and regional perfusion patterns (253).

Hypoperfusion in the occipital cortex using SPECT has also been seen in PD vs. controls (249, 254), whereas frontal lobe hypoperfusion was present in both PD and MSA-P patients (254). Hypoperfusion in the frontal lobe was observed in a 1 year longitudinal study in PD (255). In MSA-P cases, hypoperfusion in the putamen was noted compared to that in PD (256), whereas hypoperfusion together with local cerebral atrophy was found in the cerebellum and pons in MSA-C patients vs. controls (257). The current literature on perfusion SPECT is limited by lack of pathology-confirmed investigations, small sample sizes, and a handful of studies in MSA, PSP, and CBS. Moreover, given the overlap in perfusion profiles, other techniques (e.g., ^123^I-metaiodobenzylguanidine myocardial scintigraphy or DAT imaging) may perform superior to perfusion SPECT in some cases (258). Multimodal imaging can provide valuable diagnostic information in uncertain cases (10).

## Pet Imaging of Glucose Metabolism in Parkinsonian Disorders

### Metabolic Patterns Using Regional and Voxel-Based Analyses

Cerebral glucose metabolism can be evaluated using ^18^F-labeled fluorodeoxyglucose [^18^F-FDG] where reduced tracer uptake is indicative of lower glucose utilization by the tissue. Normal metabolism or hypermetabolism involving the LN (which includes putamen and globus pallidus) and possibly the thalamus, motor cortex, and cerebellum may be observed in PD on ^18^F-FDG-PET, whereas hypometabolism may be seen in parieto-occipital association areas and in the dorsolateral prefrontal cortex (259, 260). Preserved glucose metabolism in the basal ganglia may differentiate PD from MSA and PSP, where a corresponding glucose hypometabolism is typically seen in the latter (259). A meta-analysis found decreased glucose metabolism in the bilateral inferior parietal cortex and left caudate nucleus in PD, which was linked to cognitive deficits and motor symptoms, respectively (261). In MSA, glucose hypometabolism may be observed in the putamen and brainstem, with or without hypometabolism in the cerebellum compared to PD and controls (259, 262). Glucose hypometabolism may be more predominant in the bilateral putamen in MSA-P and bilateral cerebellum in MSA-C (263), based on the most affected regions in these disorders. In addition, microstructural damage as assessed using DTI was found to be associated with glucose hypometabolism in the posterior putamen in MSA-P patients (264).

In PSP, glucose hypometabolism was evident in the caudate/basal ganglia, midbrain, thalamus, as well as anterior cingulate, frontal and primary motor cortices vs. controls (259, 263, 265). Midbrain hypometabolism visualized as an oval or round region on ^18^F-FDG-PET was identified in PSP as compared to MSA and CBS patients (specificity 100%, sensitivity 29%), and may reflect midbrain atrophy (266). Compared to PD, MSA, and controls, glucose hypometabolism in PSP was reported in the caudate nucleus, thalamus, midbrain, and cingulate gyrus (262). In CBS, an asymmetric glucose hypometabolism in the basal ganglia and frontoparietal cortices may be apparent, contralateral to the clinically more affected side (263, 267). Hypometabolism in the parietal lobe may help discriminate CBS patients from PSP (268).

Patients with PDD and DLB often show a similar pattern of bilateral glucose hypometabolism vs. controls in the posterior cortical areas, including lateral frontal, temporoparietal, and occipital regions (269, 270). A more prominent hypometabolism in the anterior cingulate cortex may distinguish DLB from PDD (270). Occipital hypometabolism combined with less prominent metabolic decline in the medial temporal lobe (particularly the hippocampus) may be useful in differentiating DLB/PDD from AD (269). When differentiating DLB from AD, hypometabolism in the lateral occipital cortex achieved the highest sensitivity (88%), while relatively preserved metabolism in the posterior cingulate cortex (“cingulate island” sign) attained the highest sensitivity (100%) (271). It is suggested that ^18^F-FDG-PET may perform superior to ^123^I-IBZM-SPECT for discriminating Lewy body disorders from atypical PS (272). Moreover, computer-assisted interpretation of FDG-PET data may be used for objective evaluation in parkinsonian disorders, which can provide accuracy equivalent to visual reading especially in places where skilled readers are not available (260).

### Metabolic Patterns Based on Spatial Covariance Analysis

The spatial covariance analysis on resting-state ^18^F-FDG-PET data has enabled the identification of disease-related metabolic patterns in PD and atypical PS. In PD, a specific and relatively stable PD-related motor pattern (PDRP) and PD-related cognitive pattern (PDCP) have been identified. The PDRP is characterized by elevated pallidothalamic and pontine metabolic activity associated with reduction in the supplementary motor area, premotor cortex, and parietal association areas (273). The expression level of PDRP correlated with the loss of presynaptic nigrostriatal dopaminergic integrity and motor dysfunction (274, 275), and was also elevated in patients with idiopathic RBD (276). Likewise, the PDCP pattern was characterized by metabolic reduction in the medial frontal and parietal association regions, and metabolic increase in cerebellar cortex and dentate nuclei (273). The PDCP expression was elevated and increased over time in PD (274) and was higher in those was dementia (277). The level of PDCP expression correlated with memory and executive performance in PD (273) and, unlike PDRP, appeared relatively unaffected by treatments with intravenous levodopa or deep brain stimulation (278), which supports its utility as a reproducible imaging biomarker of cognitive dysfunction in PD.

Specific disease-related metabolic patterns based on ^18^F-FDG-PET data have also been reported for MSA, PSP (279), and CBS (267) disorders. The MSA-related pattern was elucidated as a metabolic reduction in the putamen and cerebellum, whereas PSP-related pattern showed decreased metabolism in the brainstem and medial frontal cortex compared to normal subjects (273, 279). The CBS-related pattern was characterized by asymmetric, bilateral hypometabolism involving the frontal and parietal cortex, thalamus, and caudate nucleus, where greater abnormalities were found contralateral to the clinically more affected side (267). Although, metabolic asymmetry scores for the CBS-related pattern may help in the differentiation, significant overlap with PSP patients on a case-by-case basis is often observed (267). Furthermore, the CBS-related pattern highly correlates with the features of the clinical syndrome (i.e., where) but does not provide information on the underlying causative pathology (i.e., what). In conjunction with other imaging modalities, these disease-related covariance patterns can be useful for the assessment of metabolic changes due to the underlying pathology as well as in response to disease-modifying therapies (Table 4).

**Table 4:** Summary of neuroimaging findings in α-synucleinopathies.

**Disorder, neuroimaging modality**	**Brief summary of findings^**a**^**	**References**
**Parkinson's disease**		
• Structural MRI	MRI signs: loss of dorsolateral nigral hyperintensity (swallow tail sign) ↓ in frontal lobe, basal ganglia (pronounced in advanced PD), hippocampus, anterior cingulate and superior temporal gyri, olfactory bulb and tract volume vs. NC ↓ in orbitofrontal, ventrolateral prefrontal, and occipitoparietal cortex vs. NC ↓ in olfactory bulb and tract volume in PD with olfactory disturbances vs. NC ↓ in left cuneus, precuneus, lingual gyrus and posterior cingulate cortex in PD with freezing of gait vs. PD without ↓ in right perisylvian and inferior temporal cortex along with putaminal shape changes in PD with RBD vs. PD without	(22–27, 29, 31, 39, 40, 42)
• Functional MRI	Resting-state connectivity changes in several networks (default mode, salience, central executive, sensorimotor) and in specific circuits (basal ganglia thalamocortical, cortical-subcortical sensorimotor, cerebellothalamic)	(160, 164–170)
• DWI/DTI MRI	↓ FA in SN and anterior olfactory structures and ↑*D* in olfactory tract vs. NC ↑ free water in posterior SN over time vs. NC	(52, 55–60, 64, 65)
• Proton MRS	↓ NAA/Cr ratio in SN, LN, temporoparietal and posterior cingulate cortex, and pre-SMA vs. NC	(71–74)
• PET and SPECT^b^	*Dopaminergic system:* ^b^↓ striatal presynaptic DAT binding contralateral to parkinsonian symptomatology with greater reduction in posterior putamen than in anterior putamen or caudate nucleus; normal or ↑ dopamine D2 receptor binding in drug-naïve PD vs. NC	(161, 202, 205, 206, 213, 237–240)
	*Glucose metabolism:* PD-related spatial covariance pattern may involve ↑ pallidothalamic and pontine activity associated with ↓ metabolism in SMA, premotor cortex, and parietal association areas	(273)
	*Neuroinflammation:* ↑ microglial activity in pons, basal ganglia, frontal and temporal cortex and midbrain vs. NC	(324, 325)
• Transcranial S ^b^	↑ SN echogenicity vs. HC	(179, 192)
**Lewy body spectrum disorders**		
• Structural MRI	↓ in temporoparietal and occipital cortex and in SMA in PD-MCI vs. cognitively normal PD. Diffuse atrophy in occipital, temporal, right frontal and left parietal lobe; and in putamen, hippocampus, parahippocampal region, anterior cingulate gyrus, nucleus accumbens and thalamic nuclei in PDD vs. NC. Atrophy in occipital lobe and entorhinal cortex in PDD vs. PD; in temporoparietal and occipital cortex in DLB vs. PDD. Preserved hippocampal volume (mainly cornu ammonis-1 subfield) in DLB vs. AD. *APOE*-ε4 may influence hippocampal atrophy in PDD/DLB	(8, 9, 23, 24, 79, 81, 86)
• DWI/DTI MRI	↑*D* in nucleus basalis of Meynert in PD-MCI vs. cognitively normal PD ↑*D* and ↓ FA in corpus callosum, pericallosal regions, caudate nucleus, amygdala, inferior longitudinal fasciculus, precuneus, and frontal, parietal, and occipital white matter with milder mediotemporal involvement in DLB vs. NC ↓ FA in bilateral posterior cingulate bundle in PDD vs. PD	(9, 60, 92–95)
• Proton MRS	↓ NAA/Cr ratio in hippocampus in DLB vs. NC, albeit to a lesser degree vs. AD ↓ NAA/Cr ratio in posterior cingulate gyrus in PDD vs. PD	(97, 98)
• PET and SPECT^b^	*Perfusion:* occipital hypoperfusion in DLB vs. NC	(247)
	*Glucose metabolism:* occipital hypometabolism with moderate mediotemporal hypometabolism in DLB vs. AD and HC. Hypometabolism in anterior cingulate cortex may distinguish DLB from PDD PD-related cognitive spatial covariance pattern may involve metabolic ↓ in medial frontal and parietal association regions, and metabolic ↑ in cerebellar cortex and dentate nuclei	(269, 270, 273)
	*Dopaminergic system:* ^b^ ↓ striatal presynaptic DAT binding in PDD/DLB vs. NC	(220–223)
	*Amyloid:* the gradient of increasing amyloidopathy on PET may be conceptualized as PD < PD-MCI < PDD < DLB	(281–287, 290, 291)
	*Tau:* ↑ binding in primary sensorimotor and visual cortices with less mediotemporal involvement in DLB vs. AD	(309)
	*Neuroinflammation:* ↑ microglial activity in frontal and temporal lobe, striatum, precuneus, and dorsolateral prefrontal cortex in PD-MCI vs. NC; ↑ microglial activity in anterior and posterior cingulate, striatum, and in frontal, temporal, parietal and occipital cortices with ↑ parieto-occipital binding in PDD vs. NC; the spatial extent of microglial involvement was ↑ in PDD vs. PD	(327, 330)
• Transcranial S^b^	↑ SN echogenicity in DLB and PDD vs. HC	(193)
**Multiple system atrophy**		
• Structural MRI	MRI signs: putaminal rim sign, hot cross bun sign, middle cerebellar peduncles (MCP) sign Striatonigral and olivopontocerebellar atrophy is observed in MSA vs. NC ↓ in putamen, MCP, cerebellum, and pons in MSA-P vs. PD Supratentorial atrophy (putamen) in MSA-P, whereas infratentorial atrophy in MSA-C may be more predominant	(11, 28, 71, 99, 102, 103, 106–110)
**Multiple system atrophy**		
• DWI/DTI MRI	↑*D* in putamen in MSA-P vs. PD, MSA-C, and NC. ↓ FA and ↑ ADC in putamen, cerebellum, and pons in MSA-P vs. PD and NC ↑*D* in MCP and pons in MSA vs. PSP ↑ ADC in putamen and pons in MSA-P vs. MSA-C; cerebellum and MCP in MSA-C vs. MSA-P ↑ free water-corrected FA in putamen and caudate nucleus in MSA vs. NC	(66, 113–116)
• Proton MRS	↓ NAA/Cr ratio in putamen in MSA-P, and in pontine base in both MSA-P and MSA-C vs. NC ↓ NAA/Cr ratio in putamen and pontine base may discriminate MSA-P from PD	(120)
• PET and SPECT^b^	*Glucose metabolism:* MSA-related spatial covariance pattern may involve metabolic ↓ in putamen and cerebellum	(273, 279)
	*Dopaminergic system:* ^b^ ↓ striatal presynaptic DAT binding and ↓ dopamine D2 receptor binding vs. NC	(225, 241, 242)
	*Neuroinflammation:* ↑ microglial activity in dorsolateral prefrontal cortex, putamen, pallidum, pons, and SN vs. NC	(332)
• Transcranial S^b^	↑ LN echogenicity along with normal or ↑ SN echogenicity may be seen	(188, 197)

*AD, Alzheimer's disease; ADC, apparent diffusion coefficient; D, mean diffusivity; DAT, dopamine transporter; DLB, dementia with Lewy bodies; DWI/DTI, diffusion-weighted and diffusion tensor imaging; FA, fractional anisotropy; LN, lentiform nucleus; MCP, middle cerebellar peduncle; MRI, magnetic resonance imaging; MRS, magnetic resonance spectroscopy; MSA, multiple system atrophy; MSA-C, MSA-cerebellar type; MSA-P, MSA-parkinsonian type; NAA, N-acetyl aspartate; NC, normal controls; PD, Parkinson's disease; PDD, Parkinson's disease dementia; PD-MCI, Parkinson's disease-mild cognitive impairment; PET, positron emission tomography; PSP, progressive supranuclear palsy; RBD, rapid eye movement sleep behavior disorder; SMA, supplementary motor area; SN, substantia nigra; SPECT, single photon emission computed tomography; transcranial S, transcranial sonography. a = not all findings discussed in the review are presented; please refer to relevant sections of the review for full details. b = readers are referred to Table 3 in Saeed et al. (10) for further comparisons of SPECT, PET, and transcranial sonography findings in Parkinson's disease and atypical Parkinsonian syndromes*.

## Pet Imaging of Neuropathology in Parkinsonian Disorders

### Amyloid

Cerebral amyloid deposition can be assessed on PET using ^11^C-PIB (Pittsburgh compound B) as well as using other ^18^F-labeled radiotracers (Table 2). Uptake on ^11^C-PIB PET accurately reflects amyloid deposition antemortem as validated against postmortem neuropathologic findings (280). The apparent gradient of increasing amyloidopathy as visualized on PET can be conceptualized as PD < PD-MCI < PDD < DLB, which has been supported by pathology-proven investigations (281, 282). However, inconsistencies in results may be observed in part due to the substantial pathological heterogeneity evident in these disorders.

An elevated ^11^C-PIB binding is typically observed in more than 50% of DLB cases, which can be of diagnostic relevance. Compared to controls, studies in DLB have reported elevated amyloid deposition in cortical association areas, frontal and temporoparietal cortices, cingulum and striatum, whereas non-significant differences in ^11^C-PIB binding were observed in the majority of PDD and all PD cases (283). Amyloid positivity on PET may be more frequent in PDD vs. PD cases, however, such differences were reported inconsistently (284). Compared to DLB, although most studies have found infrequent and modest uptakes in PDD cases (283), others have reported non-significant differences in binding between these two disorders (285). Similarly, a greater ^11^C-PIB retention was noted in DLB compared to PD, PD-MCI, PDD and controls—where the latter four groups showed no significant differences in the tracer retention (286). Furthermore, the *APOE*-ε4 allele has been associated with greater ^11^C-PIB binding in DLB, PDD and PD-MCI cases (286).

Elevated amyloid pathology on PET is most consistently associated with worse global cognition in LBSD, while its relationship with the timing of dementia onset, motor functions, and dementia progression is mixed (285–287). A recent 1 year study in DLB cases reported greater decline in the mini-mental state examination scores and daily functioning in those with amyloid-positive PET scan (288). In PD, the presence of striatal combined with cortical amyloidopathy was associated with greater cognitive dysfunction vs. cortical amyloidopathy alone, which underscores the cumulative and detrimental influence of amyloid deposition on cognition (289).

Greater ^11^C-PIB binding has also been associated with lower cerebrospinal fluid levels of amyloid beta-42 peptide in LBSD (290, 291). Lower medial temporal lobe perfusion was identified in amyloid-positive DLB cases (292). Glucose hypometabolism was found to align with regions of amyloid presence (293). On the other hand, ^11^C-PIB retention was virtually absent in MSA patients (293). Amyloid deposition in the brain quantified using ^11^C-PIB PET may be seen in PSP, MSA, and CBD/CBS disorders, although it may be attributable to age-related changes (294). Importantly, however, significant tracer retention on amyloid PET combined with cerebrospinal fluid findings (reduced amyloid beta-42 and elevated total tau/phospho-tau) may point toward primary Alzheimer's disease (AD) pathology, which is exclusionary to CBD/CBS and PSP diagnoses (3, 15). Thus, amyloid imaging plays a critical role in this regard and any future trials of putative tau-based disease modifying therapies that enroll patients with CBS and PSP would need to exclude AD using these methods.

### Tau

Several PET radiotracers have been developed to image tauopathy in the brain (Table 2) (295). These first-generation radiotracers initially developed for AD have generated considerable interest for their potential to quantify the topological distribution of tau, which can be valuable in monitoring disease progression and improving clinical diagnosis of PSP and CBS. However, several challenges have emerged: 1) lack of specificity and variable affinities to the multiple conformations of tau fibrils (e.g., 4R straight chain filaments in PSP vs. 3/4R paired helical filaments in AD), 2) non-negligible off-target binding to neuromelanin and monoamine oxidase A/B, and 3) the role of primary age-related tauopathy, which is not considered pathogenic and may require careful interpretation of PET data. The first-generation tau PET tracers are under study to evaluate their usefulness, and second-generation tracers with improved binding selectivity and pharmacokinetics are being developed (296).

Overall, tau PET studies reveal distinct patterns of tracer retention in tauopathies. Compared to controls, elevated ^18^F-AV-1451 uptake in PSP cases was observed in the putamen, pallidum, thalamus, midbrain, and cerebellar dentate nucleus (297–300). Likewise, increased ^18^F-AV-1451 retention in PSP was detected in the basal ganglia, although with extensive overlap and age-dependent increase in both the PSP and control groups (301). Another study included ^11^C-PIB-negative PSP patients and observed elevated ^11^C-PBB3 retention in regions similar to those seen using ^18^F-AV-1451 tracer (302). The midbrain atrophy rate, however, was identified to be a superior progression biomarker for PSP than the change in ^18^F-AV-1451 tracer retention (299). In PSP vs. PD, increased uptake in the globus pallidus, midbrain, and subthalamus was detected (303, 304). Most studies did not observe a correlation between clinical severity and ^18^F-AV-1451 uptake in PSP (299, 304), although results are mixed (300). Midbrain ^18^F-THK5351 uptake was found to correlate with clinical severity in PSP cases (305).

In CBS vs. controls, asymmetrically elevated ^18^F-AV-1451 binding contralateral to the clinically affected side was evident in the putamen, globus pallidus, and thalamus, as well as in the motor-related GM and WM subcortical structures, including the midbrain (306). Similarly, ^18^F-AV-1451 retention was observed in the motor cortex, corticospinal tract, and basal ganglia contralateral to the clinically more affected side in CBS, allowing differentiation from AD and PSP (307). However, cortical atrophy on MRI and ^18^F-FDG-PET reductions were found to be more widespread compared to the ^18^F-AV-1451 retention, making this technique a less sensitive indicator of neuronal loss (307). This observation may in part be due to the lack of ^18^F-AV-1451's specificity for 4-repeat tauopathies. Indeed, post-mortem studies have shown significant binding of ^18^F-AV-1451 to the AD-related paired helical tau filaments. Conversely, ^18^F-AV-1451 does not bind appreciably to straight tau filaments associated with 4-repeat tauopathies including PSP and CBS (297, 308). Finally, elevated ^18^F-AV-1451 binding in DLB was reported in the primary sensorimotor and visual cortices with less involvement of the temporal cortex (vs. AD), suggesting a pattern distinct from AD (309).

The ^18^F-AV-1451 tracer exhibits an off-target binding to neuromelanin neurons in the midbrain (308, 310). Compared to controls, a visually apparent decline in the midbrain ^18^F-AV-1451 signal has been reported in PD and PSP patients (310, 311), however, no correlation with disease duration or motor dysfunction was observed (298, 310). The utility of this off-target binding in differentiating PD from PSP requires further research (311). The ^18^F-AV-1451 tracer also shows an off-target binding to monoamine oxidase (MAO) A and B (312). Furthermore, recent investigations have demonstrated the binding of ^18^F-THK5351 to MAO-B—a marker for astrogliosis (313). Thus, ^18^F-THK5351 signal may suggest reactive astrocytes expressing MAO-B protein (314) and the utility of this tracer in monitoring the progression of astrogliosis in CBS has been proposed (315). Overall, an off-target binding to MAO (and other targets) can confound PET data, especially given the abundance of these proteins across the entire brain, which can fluctuate during the course of the disease and with treatment interventions.

Recently, it is suggested that ^11^C-PBB3 and ^18^F-AV-1451 radioligands may show differential selectivity for the different tau isoforms, with ^18^F-AV-1451 binding predominantly to AD-type tau deposits, whereas ^11^C-PBB3 and the THK family of tracers exhibiting a higher affinity for non-AD tau aggregates (i.e., 4R as seen in CBD/PSP) (316, 317). Moreover, besides binding tau aggregates in PSP, the ^11^C-PBB3 tracer also showed binding in patients expected to have α-synuclein pathology (318). It was suggested that ^11^C-PBB3 may exhibit off-target binding to α-synuclein or other associated proteins (318), although further studies are required for confirmation. Indeed, given the conformational diversity of tau fibrils, validating ligands specific to different tau strains would be promising in the differential diagnosis of tauopathies, which can be challenging to diagnose at early stages.

### Alpha-Synuclein

Successful imaging of α-synuclein pathology using PET radiotracers is expected to be transformative in clinical and research settings. Several radiolabeled probes for imaging α-synucleinopathy in the brain have been explored, including the phenothiazine, indolinone, indolinone-diene and chalcone analogs, and structural congeners (319). However, no radiotracer has currently been approved for use in humans for diagnostic or research purposes. A consortium of researchers has been convened by the Michael J. Fox Foundation to develop α-synuclein PET radiotracers. When developed, these tracers may allow, (1) the identification of patients at prodromal or early stages of an α-synucleinopathy, (2) evaluation of the degree, location, and progression of the disease, as well as therapeutic effectiveness, (3) differentiation of α-synucleinopathies (LBSD and MSA) from tauopathies (PSP and CBD) and AD, and (4) insights into the contribution of α-synuclein pathology for clinical outcomes (320). Some of the developmental challenges [see reference (321)], although similar to those of tau PET radiotracers, include the intracellular nature of most α-synuclein aggregates requiring ideal lipophilicity and molecular size, multiple α-synuclein strains that may interact differently with some tracers, colocalization of α-synuclein with other protein aggregates, relatively less abundance of α-synuclein over amyloid and tau aggregates requiring higher tracer selectivity, as well as the potential for off-target binding which may necessitate further validation.

## Pet Imaging of Neuroinflammation in Parkinsonian Disorders

Microglia are the primary macrophages involved in the innate immune response of the central nervous system. It is suggested that microglia-mediated inflammatory processes can aggravate injury, leading to events that may result in neurodegeneration (322). A widely used PET ligand for imaging neuroinflammation has been ^11^C-PK11195, which binds to the 18 kDa translocator protein (TSPO), located on the outer mitochondrial membrane in microglia. Upregulation of TSPO is suggestive of microglial activation in the central nervous system. However, there are several limitations of ^11^C-PK11195 (e.g., non-specific binding, low brain penetration, high plasma protein binding), which has prompted the development of improved second-generation radiotracers, including ^11^C-PBR28 and ^18^F-FEPPA (323).

Compared to controls, increased ^11^C-PK11195 binding in PD was observed in the pons, basal ganglia, frontal and temporal cortices (324), as well as in the midbrain contralateral to the clinically affected side (325). The correlation of ^11^C-PK11195 binding with clinical severity or with putaminal presynaptic dopaminergic integrity is inconsistently reported (324, 325). Recently, considerable interindividual variability in the binding affinity of second-generation radiotracers (e.g., ^18^F-FEPPA) to the TSPO protein has been observed, which is attributed to a single nucleotide polymorphism located at exon 4 of the *TSPO* gene (rs6971) (323, 326). Three patterns of binding affinities based on this genetic polymorphism have been identified: low, high, and mixed affinity binders. This finding suggests a possible interaction between the rs6791 polymorphism and neuroinflammation resulting in interindividual variability in the outcome measures. Further studies are warranted to study this interaction. Increased ^18^F-FEPPA binding was also reported in PD-MCI in the frontal and temporal lobe, striatum, precuneus, and dorsolateral prefrontal cortex in association with amyloid deposition in these regions (327), suggesting a link between amyloidopathy and neuroinflammation.

RBD is a significant risk factor for the development of α-synucleinopathies (236). In polysomnography-confirmed cases with idiopathic RBD, elevated ^11^C-PK11195 binding was reported in the occipital lobe (probably highlighting those at increased risk of developing DLB) (328), as well as in the left SN, although no significant differences in ^11^C-PK11195 binding were noted in the putamen or caudate (despite lower putaminal ^18^F-dopa uptake) (329). Longitudinal studies are needed to identify biomarkers for those likely to convert earlier vs. later into an overt symptomatic synucleinopathy (i.e., dementia or parkinsonism) when presenting with RBD.

PDD patients exhibited widespread microglial activation vs. controls in the anterior and posterior cingulate, striatum, as well as in the frontal, temporal, parietal, and occipital cortices, with pronounced parieto-occipital binding (330). Compared to PD, however, the spatial extent of microglial cortical involvement was greater in PDD (330). Additionally, an inverse correlation between microglial activation and glucose metabolism in the temporoparietal cortex has been reported in PDD (331). As consistent with the known neuropathological burden, augmented ^11^C-PK11195 binding compared to controls was evident in the dorsolateral prefrontal cortex, putamen, pallidum, pons, and SN in MSA cases (332), and in the basal ganglia, midbrain, frontal lobe, and cerebellum in PSP cases (324). Microglial activity in PSP-RS was identified in the thalamus, putamen, and pallidum (333). Finally, CBS patients typically show ^11^C-PK11195 uptake in the caudate nucleus, putamen, SN, pons, pre- and post-central gyrus and the frontal lobe (334). Although, the overall patterns of microglial activity align with neuropathological findings, these patterns may not allow differentiation in a clinical setting, and may be more appropriate as biomarkers of prodromal changes or therapeutic effectiveness in clinical trials.

Summary of neuroimaging findings in α-synucleinopathies and tauopathies are presented (Tables 4, 5).

**Table 5:** Summary of neuroimaging findings in tauopathies.

**Disorder, neuroimaging modality**	**Brief summary of findings^**a**^**	**References**
**Progressive supranuclear palsy**		
• Structural MRI	MRI signs: hummingbird sign, morning glory sign ↓ in midbrain and SCP vs. PD, MSA-P, CBD/CBS, and NC ↓ in brainstem and gray matter of frontal lobe vs. NC Magnetic resonance parkinsonism index: ratios of pons-to-midbrain area and MCP-to-SCP widths were ↑ in PSP vs. PD, MSA-P and NC. The revised version of index incorporates third ventricle width and frontal horns width	(121–129, 131, 132, 136)
• DWI/DTI MRI	↑*D* and ↓ FA in multiple regions including midbrain, SCP, orbitofrontal white matter, thalamus, motor and SMA vs. NC ↑*D* in the decussation of SCP vs. MSA and PD ↑ ADC in putamen, globus pallidus, and caudate nucleus vs. PD ↓ FA in SCP in PSP-RS vs. PSP-parkinsonism ↑ free water in posterior SN vs. MSA, PD and NC; ↑ free water-corrected FA in caudate nucleus, putamen, thalamus and vermis and ↓ in SCP and corpus callosum vs. NC	(66, 115, 118, 132, 139–142, 145–147)
• Proton MRS	↓ NAA/Cr ratio in LN, brainstem, centrum semiovale, frontal, and precentral cortex vs. NC ↓ NAA/choline ratio in LN vs. NC; ↓ NAA/Cr ratio in putamen vs. PD and MSA ↓ cerebellar NAA/Cr and NAA/myo-inositol ratios in PSP-RS vs. NC, and ↓ cerebellar NAA/Cr ratio in PSP-RS vs. PD	(77, 148–150)
• PET and SPECT^b^	*Dopaminergic system:* ^b^ ↓ striatal presynaptic DAT binding, with ↑ but fairly uniform DAT loss in striatum vs. PD ↓ dopamine D2 receptor binding vs. NC	(200, 204, 229, 230, 239, 242)
	*Glucose metabolism:* PSP-related spatial covariance pattern may show hypometabolism in brainstem and mediofrontal cortex	(273, 279)
	*Tau:* ↑ binding in putamen, pallidum, thalamus, midbrain, cerebellar dentate nucleus, and basal ganglia vs. NC ↑ binding in globus pallidus, midbrain, and subthalamus in PSP vs. PD	(297–301, 303, 304)
	*Neuroinflammation:* ↑ microglial activity in basal ganglia, midbrain, frontal lobe, and cerebellum vs. NC	(324)
**Corticobasal degeneration/syndrome**		
• Structural MRI	Atrophy patterns align with the “true” underlying pathology The predominant clinical syndrome in CBS relates closely to regional atrophy patterns In general, asymmetric cortical atrophy in frontoparietal lobe contralateral to more affected side in CBD/CBS vs. NC ↓ in bilateral premotor cortex, superior parietal lobules, and striatum in CBS vs. NC ↓ in dorsofrontal and parietal cortex and ↑ global brain atrophy in CBS vs. PSP; ↓ in midbrain in PSP vs. CBS	(6, 121, 123, 129, 152, 153, 155, 156)
• DWI/DTI MRI	↑*D* and ↓ FA in posterior truncus of corpus callosum in CBS vs. PD and NC ↓ FA in long frontoparietal connecting tracts, intraparietal associative fibers, corpus callosum, and sensorimotor cortical projections in CBS vs. NC	(59, 152, 158)
• Proton MRS	↓ NAA/Choline and NAA/Cr ratios in contralateral frontoparietal cortex, LN and centrum semiovale in CBS vs. NC ↓ NAA/Cr ratio in frontal cortex and asymmetrically in putamen in CBS vs. PD, MSA and vascular parkinsonism	(149, 150, 159)
• PET and SPECT ^b^	*Glucose metabolism:* CBS-related spatial covariance pattern may show asymmetric, bilateral hypometabolism involving frontal and parietal cortex, thalamus, and caudate nucleus, with ↑ abnormalities contralaterally	(267)
	*Dopaminergic system:* ^b^ ↓ striatal presynaptic DAT binding with hemispheric asymmetry vs. PD.	(226, 227)
	*Tau:* ↑ asymmetric binding contralateral to the clinically affected side in putamen, globus pallidus, thalamus, and midbrain vs. NC, and in motor cortex, corticospinal tract, and basal ganglia vs. AD and PSP	(306, 307)
	*Neuroinflammation:* ↑ microglial activity in caudate nucleus, putamen, SN, pons, pre- and post-central gyrus and frontal lobe vs. NC	(334)

*AD, Alzheimer's disease; ADC, apparent diffusion coefficient; APOE-ε4, apolipoprotein E ε4-allele; CBD, corticobasal degeneration; CBS, corticobasal syndrome; NC, normal controls; Cr, creatine; D, mean diffusivity; DAT, dopamine transporter; DWI/DTI, diffusion-weighted and diffusion tensor imaging; FA, fractional anisotropy; LN, lentiform nucleus; MCP, middle cerebellar peduncle; MRI, magnetic resonance imaging; MRS, magnetic resonance spectroscopy; MSA, multiple system atrophy; MSA-P, MSA-parkinsonian type; NAA, N-acetyl aspartate; PD, Parkinson's disease; PET, positron emission tomography; PSP, progressive supranuclear palsy; PSP-RS, PSP-Richardson's syndrome; SCP, superior cerebellar peduncle; SMA, supplementary motor area; SN, substantia nigra; SPECT, single photon emission computed tomography. a = not all findings discussed in the review are presented; please refer to relevant sections of the review for full details. b = readers are referred to Table 3 in Saeed et al. (10) for further comparisons of SPECT, PET, and transcranial sonography findings in Parkinson's disease vs. atypical Parkinsonian syndromes*.

## Conclusions

Research using multimodal neuroimaging has facilitated a better understanding of the disease processes in PD and atypical PS—neurodegenerative disorders that often present with substantial clinical and pathological heterogeneity. As per the “multimodal approach,” multiple biomarkers obtained from different neuroimaging modalities can provide distinct yet corroborative data on the underlying neurodegenerative processes, and this integrative approach may prove superior to single-modality-based methods. Future efforts are needed to validate biomarkers in well-characterized cohorts. Some of these biomarkers may help improve the current consensus diagnostic guidelines and allow clinicians to ascertain an optimal approach for diagnostic purposes in combination with their experience and professional training.

## Author Contributions

US reviewed the literature, wrote the manuscript, and prepared tables and figures. MM supervised the writing of this review paper. MM and AL reviewed and critiqued the manuscript for intellectual content. All authors read and approved the final manuscript.

## Conflict of Interest

The authors declare that the research was conducted in the absence of any commercial or financial relationships that could be construed as a potential conflict of interest.

## Abbreviations

AADC: Aromatic amino acid decarboxylase
AD: Alzheimer's disease
ADC: Apparent diffusion coefficient
APOE-ε4: Apolipoprotein E ε4-allele
CBD: Corticobasal degeneration
CBS: Corticobasal syndrome
Cr: Creatine
*D*: Mean diffusivity
DAT: Dopamine transporter
DLB: Dementia with Lewy bodies
DTI: Diffusion tensor imaging
FA: Fractional anisotropy
GM: Gray matter
LBSD: Lewy body spectrum disorders
LN: Lentiform nucleus
MAO: Monoamine oxidase
MCP: Middle cerebellar peduncle
MRI: Magnetic resonance imaging
MSA: Multiple system atrophy
MSA-C: Multiple system atrophy-cerebellar type
MSA-P: Multiple system atrophy-parkinsonian type
NAA: N-acetyl aspartate
NPV: Negative predictive value
PD: Parkinson's disease
PDD: Parkinson's disease dementia
PD-MCI: Parkinson's disease-mild cognitive impairment
PDRP: Parkinson's disease-related motor pattern
PDCP: Parkinson's disease-related cognitive pattern
PET: Positron emission tomography
PPV: Positive predictive value
PS: Parkinsonian syndrome
PSP: Progressive supranuclear palsy
PSP-RS: Progressive supranuclear palsy-Richardson's syndrome
RBD: Rapid eye movement sleep behavior disorder
SCP: Superior cerebellar peduncle
SN: Substantia nigra
SNpc: Substantia nigra pars compacta
SPECT: Single photon emission computed tomography
TDP-43: Transactive response DNA binding protein-43 kDa
TSPO: Translocator protein-18 kDa
VBM: Voxel-based morphometry
VMAT2: Vesicular monoamine transporter type 2
WM: White matter.
